# Therapeutic effect and mechanism of gigantol on hyperuricemia

**DOI:** 10.3389/fendo.2025.1474808

**Published:** 2025-07-29

**Authors:** Yuanfan Wu, Xia Sun, Yuhan Jia, Tianshu Gao, Jin Xu, Youqiao Qian, Naiqi Pei, Lilin Wang, Qiaohong Zheng, Honglei Li, Zhen Chen, Yijiao Liu, Yang Ma, Hui Chen, Yuanyuan Ye, Jiaxin Zhao, Yi Zhou, Xiaoqing Chen, Baosheng Huang, Yefeng Liu, Yin Zhu, Ning Xue, Juan Zhang, Guangfeng Ji, Xing Wang

**Affiliations:** ^1^ Department of Geriatrics, The First Affiliated Hospital of Nanjing Medical University, Nanjing, China; ^2^ Department of Medicine, Ju Rong Hospital of Traditional Chinese Medicine, Zhenjiang, Jiangsu, China; ^3^ School of Pharmacy, China Pharmaceutical University, Nanjing, China; ^4^ Department of Nephrology, Jurong Hospital Affiliated to Jiangsu University, Jurong, China; ^5^ College of Mudi Meng Honors, China Pharmaceutical University, Nanjing, China; ^6^ School of Basic Medicine and Clinical Pharmacy, China Pharmaceutical University, Nanjing, China; ^7^ College of Life Science and Technology, China Pharmaceutical University, Nanjing, China; ^8^ Department of Pharmacy, Kangda College of Nanjing Medical University, Lianyungang, China; ^9^ Department of Neurosurgery, Sir Run Run Hospital, Nanjing Medical University, Nanjing, China; ^10^ Department of General Surgery, Jurong Hospital Affiliated to Jiangsu University, Jiangsu, Zhenjiang, China; ^11^ Department of General, Jurong Hospital Affiliated to Jiangsu University, Jiangsu, Zhenjiang, China; ^12^ Department of Acupuncture, Jurong Hospital Affiliated to Jiangsu University, Jiangsu, Zhenjiang, China; ^13^ The Fourth Department of Psychiatry, Shandong Daizhuang Hospital, Shandong, Jining, China

**Keywords:** gigantol, HUA, xanthine oxidase inhibitor, UA transporters, NLRP3

## Abstract

This study aimed to evaluate the therapeutic effects of gigantol on hyperuricemia (HUA) and investigate the underlying mechanism of HUA. A mouse model of HUA was made by gavage of potassium oxonate, and HK-2 and AML12 cell models were made by adenosine and xanthine oxidase (XOD) induction. We tested the levels of uric acid (UA), creatinine (CRE), blood urea nitrogen (BUN), cellular UA, and XOD activity. The levels of NOD-like receptor thermal protein domain 3 (NLRP3) and other inflammatory factors were detected by enzyme-linked immunosorbent assay (ELISA) kits. XOD is a protein related to the NLRP3 pathway and also serves as an UA transporter. We found that the levels of UA, CRE, and BUN increased in serum but decreased in urine in HUA model mice. After gigantol treatment, UA, CRE, and BUN levels in serum decreased, whereas their levels in urine increased. The levels of NLRP3 and interleukin-1β (IL-1β) were lower and the expression of NLRP3-related protein decreased after gigantol treatment. In conclusion, gigantol exhibits a therapeutic effect on HUA, and the mechanism may be related to inhibiting XOD activity to reduce UA production, regulating the expression of UA transporters to increase UA excretion, and inhibiting the activation of NLRP3 inflammatory signaling.

## Highlights

- Gigantol has a good therapeutic effect on hyperuricemia.
- Gigantol reduces uric acid by inhibiting XOD activity and restoring normal expression of uric acid transporters.
- Hyperuricemia is associated with the NLRP3 inflammatory signaling pathway, and gigantol reduces the inflammatory response.

## 1 Introduction

Hyperuricemia (HUA) refers to a higher uric acid (UA) level than normal in the state of a normal purine diet. The normal UA value is less than 420 μmol/L in men and below 360 μmol/L in women. Either excessive UA secretion or decreased UA excretion leads to HUA (1). HUA is a disease characterized by increased UA in the blood due to the impairment of purine metabolism and/or the decrease of excretion of UA in the body, with a high rate of onset in old men and postmenopausal women. With an increase in standard of living and a change in lifestyle, the incidence of HUA shows an increasing trend, even at a younger age. The most common clinical manifestation of HUA is gout, which affects patients’ physical and mental health and quality of life. Although HUA is not classified as a fatal disease, it can induce many major diseases, such as myocardial infarction, coronary heart disease, diabetes mellitus, hyperlipidemia, metabolic syndrome, and chronic kidney disease.

HUA is mainly associated with UA overproduction and UA excretion deficiency. HUA can be divided into two main categories: Primary HUA, mainly resulting from inborn errors of purine metabolism or molecular defects of some cause, is often accompanied by one or more metabolic syndromes of different categories; secondary HUA is mainly caused by certain diseases or drugs. Reducing serum UA levels is central to the control of HUA. Currently, the drugs used in clinical practice mainly reduce UA in three ways: inhibiting UA synthesis, promoting UA excretion, and promoting UA breakdown.

Xanthine and hypoxanthine in the body can be oxidized by xanthine oxidase (XOD) to produce UA and xanthine. Inhibition of XOD activity can reduce the production of UA in the body and reduce the concentration of UA in the blood; thus, XOD has currently become an important target for the development of urate-lowering drugs. Allopurinol is a widely used first-line inhibitor of UA production. Allopurinol is an isomer of hypoxanthine that competitively binds to the active site of XOD and inhibits the metabolism of hypoxanthine to UA (2). However, long-term use of allopurinol is prone to cause serious adverse effects, such as leukopenia thrombocytopenia, eosinophilia, and fever. Febuxostat is a UA-lowering drug that has ushered in a new era of gout treatment, especially for patients intolerant to allopurinol. However, long-term use of febuxostat could cause liver and kidney injury. Moreover, febuxostat is more expensive compared with allopurinol. Therefore, more potent, less toxic, and cheaper urate-lowering drugs should be urgently developed.

A previous study showed that *Dendrobium candidum* has the effect of reducing UA (3), but the specific bioactive compound responsible for this effect remains unclear. Gigantol, a biphenyl compound primarily extracted from *Dendrobium* (4, 5), has been reported to possess analgesic, anti-inflammatory, immunomodulatory, hypoglycemic, and antitumor properties (6–10). However, the rapid metabolism and clearance of gigantol may limit its clinical applicability, as frequent dosing would be required to maintain effective concentrations (11). In contrast, allopurinol and its active metabolites have a longer half-life, allowing for once-daily administration. To improve the feasibility of gigantol, further research on sustained-release formulations or structural modifications to prolong its duration of action is warranted, alongside an evaluation of its safety–efficacy balance. To date, the effect and mechanism of gigantol on UA reduction in patients with HUA remain unreported.

The inflammasome of NOD-like receptor thermal protein domain 3 (NLRP3) is a protein complex composed of a NOD-like receptor, an apoptosis-related dot-like protein (ASC), and aspartate specific protease 1 (caspase-1) (12). Urate crystals can act on NLRP3 receptors to promote the expression of proinflammatory cytokines and chemokines, including interleukin-1β (IL-1β) and interleukin-6 (IL-6), which are involved in inflammatory and fibrotic pathologies (13). NLRP3 overexpression accelerates HUA progression, suggesting that NLRP3 inflammasome may be a new therapeutic target for HUA.

Normally, 70% of UA is excreted by the kidneys (14), and reduced excretion of UA is mostly associated with dysfunction of renal urate transport (15). Urate transporters are mainly classified into two groups: urate secretory proteins and urate reabsorption proteins. Organic anion transporter 1 (OAT1) and organic anion transporter 3 (OAT3) are key proteins involved in the secretion of UA, which are distributed in the basal lateral of proximal tubular epithelial cells. OAT1 and OAT3 play the role of an organic anion/ketoglutarate exchanger, complete the uptake of UA while discharging α-ketoglutaric acid, and reduce the content of UA in the blood. UA reabsorption proteins include recombinant urate transporter 1 (URAT1) and human glucose transporter 9 (GLUT9). URAT1 is distributed on the lumen side or the outside of the basement membrane of epithelial cells in the proximal tubules. URAT1 mediates the exchange of UA in the lumen with inorganic and organic anions in the proximal tubular epithelial cells, thus promoting the transport of UA into epithelial cells. GLUT9 is located in the basolateral and apical side of proximal tubule epithelial cells, and intracellular UA is translocated to the renal interstitium via GLUT9, which promotes the reabsorption of UA from urine into blood (16).

The HK-2 cell line (human renal proximal tubular epithelium) was selected based on its well-established relevance in urate transport research. Recent studies (17) have confirmed that HK-2 cells consistently express pivotal urate transporters including URAT1 and GLUT9, mirroring the pathophysiological alterations observed in renal tubular epithelia of chronic kidney disease patients.

For the AML12 murine hepatocyte model, our selection criteria emphasize its physiological representation of hepatic urate biosynthesis. Preliminary validation data demonstrate that AML12 cells maintain stable XOD activity—the rate-limiting enzyme in UA production. Importantly, this non-transformed hepatic cell line retains characteristic metabolic features of normal hepatocytes, unlike tumor-derived models (e.g., HepG2), thereby providing superior translational relevance for studying purine metabolism homeostasis.

Therefore, in this study, we established *in vivo* and *in vitro* HUA models to investigate whether gigantol, an important component of *Dendrobium officinale*, has a therapeutic effect on HUA and explore the underlying mechanism by examining NLRP3 signaling pathway and urate transporters.

## 2 Materials and methods

### 2.1 Reagents

Gigantol (5-[2-(3-hydroxy-5-methoxyphenyl) ethyl]-2-methoxyphenol, CAS: 67884-30-4), a natural product derived from *Dendrobium* with 98% purity, was purchased from Acmec Biochemical (Shanghai, China). Fetal bovine serum (FBS), phosphate-buffered saline (PBS), BCA protein assay kit, 4% paraformaldehyde solution, mouse IL-1β, IL-6, and NLRP3 enzyme-linked immunosorbent assay (ELISA) kits (assay range: 0.8–35 pg/mL) were all purchased from Senbega Biotechnology (Nanjing, China). The detection kits of UA, blood urea nitrogen (BUN), creatinine (CRE), and XOD were obtained from Nanjing Jiancheng Biotechnology Institute (Nanjing, China). Potassium oxonate and allopurinol were purchased from Aladdin Biochemical Technology Co., Ltd (Shanghai, China). Adenosine and XOD were purchased from Sigma (St. Louis, MO, USA). RPMI-1640 and DMEM/F12 medium were acquired from Thermo Fisher (Grand Island, NY, USA).

### 2.2 Bioinformatics analysis

The potential molecular targets of gigantol were predicted using the Swiss Target Prediction (18) and the similarity ensemble approach (SEA) database (19). An online software STRING was used to obtain PPI data (20). The online software generates a score for each protein interaction information. The higher the score, the greater the credibility of the interaction between the target proteins. Therefore, in the parameter setting, we have selected the *Mus musculus*, and the confidence value is set to medium confidence (0.4000).

Gene Ontology (GO) enrichment analysis and Kyoto Encyclopedia of Genes and Genomes (KEGG) pathway enrichment analysis were carried out to further study the functions of the identified potential anti-HUA target genes of gigantol based on R 4.0.2 and related R packages (colorspace, stringi, DOSE, clusterProfiler, ggplot2, enrichplot, pathview, BiocManager, and org.Hs.Mm.db).

The network was constructed using Cytoscape 3.8.2 software. The constructed component, target, and signaling pathway network were constructed based on the active components obtained from the analysis, the predicted target proteins, and signaling pathways related to HUA. The nodes in the network diagram represent the chemical components analyzed or the predicted target proteins and signaling pathways, and the edges represent the compound–target or target–pathway interactions.

### 2.3 Animal experiments

All experimental animals were performed in accordance with the regulations of the Jiangsu Province laboratory animal ethics committee, and the experimental protocols were approved by the animal care and ethics committee of China Pharmaceutical University. SPF male KM mice (6–8 weeks old, 20 ± 2 g) were purchased from Jiangsu Huachuang Xinnuo Pharmaceutical Technology [Permit ID: SCXK (SU) 2020-0005]. Animals were kept under a normal 12-h/12-h light/dark schedule. They were housed at room temperature (22 ± 2°C) with relative humidity (55% ± 5%) and given a standard chow and water *ad libitum*.

After mice were adaptively fed for 7 days, 48 mice were randomly assigned to six groups (eight mice per group) as follows: blank, model, allopurinol (10 mg/kg), low dose of gigantol (5 mg/kg), medium dose of gigantol (10 mg/kg), and high dose of gigantol (20 mg/kg).

Animal grouping and experimental procedures were conducted in accordance with ARRIVE guidelines 2.0 (21). Rats were randomly allocated using stratified randomization based on weight levels (R package “blockrand”). Treatment administration and outcome assessment were performed by different investigators blinded to group assignments. All biochemical analyses were conducted with sample codes obscured.

Mice in all groups except the blank control group were intragastrically administered potassium oxonate (300 mg/kg) at a dose of 10 mL/kg. The blank control group was gavaged with an equal volume of normal saline.

Rationale for the dosage of potassium oxonate: The selection of the 300 mg/kg dosage of potassium oxonate is based on several factors. Firstly, this dose has been well-established in previous studies to effectively inhibit the activity of enzymes related to UA metabolism, such as XOD, in mice. It can increase UA production or decrease its excretion, thereby inducing a stable and distinct HUA state. Considering the strain and weight range of the experimental mice, this dosage has been proven to be suitable for successfully replicating the HUA model. Secondly, using this dose facilitates comparison and verification with other research results, ensuring the consistency and reproducibility of experimental outcomes in the academic field.

One hour after potassium oxonate administration, the corresponding drugs in each group were administrated by gavage for 7 days continuously (22).

Rationale for the time interval of 1 h: The 1-h interval between potassium oxonate administration and subsequent drug administration is determined through preliminary pre-experiments and references. After intragastric administration, potassium oxonate requires a certain period for absorption and distribution in mice to reach a relatively stable concentration in the blood and tissues that can exert its modeling effect. At the 1-h time point, potassium oxonate has basically completed the initial absorption process, and its blood concentration is relatively stable. At this moment, administering the subsequent drugs allows them to more accurately exert their effects on the basis of the HUA model. If the interval is too short, potassium oxonate may not be fully absorbed, leading to an unstable HUA model and inaccurate evaluation of the subsequent drug effects. If the interval is too long, potassium oxonate may be metabolized in the body, and its blood concentration may decrease, which also affects the stability and consistency of the HUA model and is not conducive to accurately studying the intervention effects of subsequent drugs on HUA.

On day 6, the mice were transferred to a metabolic cage, given free water and diet, and 24-h urine samples were collected for the determination of UA, CRE, and BUN levels. One hour after the last administration, 2 mL of blood was taken through eyeball extraction, and the mice were killed by euthanasia. Mice were dissected and the livers and kidneys were removed.

### 2.4 Biochemical analysis

Blood obtained from mice in each group was centrifuged at 3,000 r/min for 15 min in a low-temperature high-speed centrifuge. Then, the serum was separated, and the levels of UA, CRE, and BUN in the serum were measured by the kits. The levels of TNF-α, IL-1β, IL-6, and NLRP3 in serum were detected by ELISA kits. Liver and kidney tissues were homogenized by adding ice-cold saline, and homogenates were centrifuged at 12,000 r/min for 10 min. XOD activity in the supernatant of the liver tissues was measured, and the levels of TNF-α, IL-1β, IL-6, and NLRP3 in the supernatant of the kidney tissues were measures by ELISA kits.

### 2.5 HE staining

The kidney and liver tissues were fixed with 4% paraformaldehyde solution. After paraffin embedding and sectioning, the paraffin on the slide was removed with xylene and soaked in different concentrations of ethanol and deionized water. The liver and kidney tissues were stained according to the HE staining procedure, and then the morphology of the kidney tissues and liver tissues was observed under the microscope.

### 2.6 Western blot analysis

Proteins were extracted from tissues or cells using RIPA lysis buffer. The protein concentration was determined using a BCA protein assay kit. Equal amounts (30 μg) of proteins were separated by sodium dodecyl sulfate polyacrylamide gel electrophoresis (SDS-PAGE) and transferred to polyvinylidene fluoride (PVDF) membranes. The membranes were blocked with 5% non-fat milk for 2 h at room temperature, then washed in Tris-HCl Tween (TBST) three times (5 min for each). The membranes were incubated with the following primary antibodies at 4°C overnight (ASC, 1:1,000; caspase-1, 1:2,000; NLRP3, 1:2,000; GLUT9, 1:2,000; OAT1, 1:2,000; OAT3, 1:2,000, and β-actin, 1:5,000). The membranes were washed in TBST solution three times and incubated with secondary antibodies (1:10,000 dilution) for 2 h at 37°C. After three washes (5 min each) in TBST solution, protein bands were detected by the ECL kit and analyzed by ImageJ software.

### 2.7 Cell culture

Alpha mouse liver 12 (AML12) and human renal proximal tubule (HK-2) cells were provided by the Cell Bank of the Chinese Academy of Sciences (Shanghai, China). HK-2 cells were cultured in RPMI 1640 medium supplemented with 10% FBS, 100 μg/mL streptomycin, and 100 units/mL penicillin while AML12 cells were cultured in DMEM complete medium.

### 2.8 CCK8 assay

AML12 cells and HK-2 cells were plated at a density of 1.0×10^4^ cells/well in 96-well plates. After incubation for 24 h, cells were treated with gigantol at 1,000, 500, 250, 125, 62.5, and 0 μmol/L for 24 h. 10% CCK8 was added to each well, and after incubation for 4 h, the absorbance at 450 nm was measured.

### 2.9 HUA cell model

The HK-2 cell HUA model was established according to reported methods (23). HK-2 cells were exposed to adenosine solution with the concentration of 2.5 mmol/L for 24 h. Then, XOD (0.005 IU/mg) was added into each hole of the 24-well plates, incubated for 8 h, and the supernatant was collected to measure the UA level using the UA kit.

The *in vitro* model of HUA in AML12 cells was similar except that the concentration of adenosine solution was 1.5 mmol/L.

### 2.10 Assay of UA levels in AML12 cells and HK-2 cells

AML12 cells and HK-2 cells were cultured in DMEM and RPMI 1640 complete medium, respectively, at 37°C under 5% CO_2_. Logarithmically growing cells were seeded into 24-well plates at a concentration of 1×10^5^ cells/mL and incubated for 24 h under the same conditions. When the cells reached 80% confluency, they were washed three times with PBS. In the experimental group, cells were treated with gigantol (25, 50, and 100 μmol/L) prepared in serum-free medium. For the positive control, allopurinol (100 μmol/L) prepared in serum-free medium was used, and the blank control group and the model group were added with serum-free medium. After 24-h incubation, the cells were rinsed three times with PBS. The model group, the experimental group, and the positive control group were treated with 2.5 mmol/L adenosine solution prepared in serum-free medium for 24 h. Except for the blank group, XO solution was added to each well. The supernatants were collected after 12 h of incubation to detect UA levels in AML12 and HK-2 cells as recommended by the UA test kit manufacturer.

### 2.11 Assay of inflammatory factors in HK-2 cells

HK-2 cells were treated as described above. The supernatant of the medium was collected, and the levels of proinflammatory cytokines (TNF-α, IL-6, and IL-1β) and the NLRP3 inflammasome component in the supernatant were detected using commercially available ELISA kits according to the manufacturers’ protocols.

### 2.12 Pharmacokinetics of gigantol and allopurinol in rats

Twelve SD rats were randomly divided into four groups (*n* = 3 per group): the gigantol gavage group (2 mg/mL), the allopurinol gavage group (2 mg/mL), the gigantol intravenous group (0.5 mg/mL), and the allopurinol intravenous group (0.5 mg/mL). In the intravenous group, the rats received intravenous gigantol and allopurinol at doses of 2 and 5 mg/kg, respectively. Whole blood was taken into heparinized tubes at predefined time points (2, 5, 10, 20, and 30 min, and 1, 2, 4, 6, 8, and 12 h) after administration, and plasma was separated by centrifugation and stored at −20°C until testing. The rats in the gavage group were administrated with gigantol and allopurinol at doses of 20 and 50 mg/kg by gavage. Whole blood was taken into heparinized tubes at predefined time points (2, 5, 10, 20, and 30 min, and 1, 2, 4, 6, 8, and 12 h) after administration, and plasma was separated by centrifugation and stored at −20°C until testing. The concentrations of gigantol and allopurinol in rat plasma were quantified by the LC-MS/MS method. Pharmacokinetic parameters were calculated by using the pharmacokinetics software WinNonlin 8.1.

### 2.13 Molecular docking of gigantol

The molecular docking method was used to verify the action site and pharmacological effect of gigantol. Molecular docking protein crystal XOD (PDB ID:1N5X) (24) and NLRP3 (PDB ID:7ALV) (25) were downloaded from the Protein Data Bank (PDB; https://www.rcsb.org/). The amino acid sequences of hGLUT9 (UniProt ID: Q9NRM0), OAT1 (UniProt ID: Q4U2R8), and OAT3 (UniProt ID: Q8TCC7) were obtained from the UniProt database (https://www.uniprot.org/). Protein modeling was based on the AlphaFold Protein Structure Database (https://alphafold.ebi.ac.uk/) (26, 27). The active sites of XOD (PDB ID:1N5X) were determined by the original ligand febuxostat, and those of hGLUT9, OAT1, and OAT3 were determined by methods reported previously (28, 29). Software AutoDock 4.0 was used for molecular docking, and PyMOL software was used for plotting.

### 2.14 Statistical analysis

Sample size justification was performed via *post-hoc* power analysis using G*Power 3.1. Based on the observed effect sizes (Cohen’s *d* = 2.06 for treatment groups vs. model group) and a significance level of α = 0.05, the statistical power exceeded 96.5% for high-dose comparisons, and a *post-hoc* power analysis (G*Power 3.1) for the one-way analysis of variance (ANOVA) indicated a statistical power of 100% (α = 0.05, Cohen’s *f* = 2.18), confirming that the sample size (*n* = 8 per group) was sufficient to detect overall differences among the six groups. All results were presented as mean ± SEM. For normally distributed data, *p*-values were calculated using Student’s *t*-test. When three or more groups were analyzed, one-way ANOVA with *post-hoc* Tukey’s test was used to assess the differences.

## 3 Results

### 3.1 Identification of HUA-related targets of gigantol

Based on the chemical structure of gigantol, 49 putative targets were obtained from the Swiss Target Prediction and 68 targets were obtained from SEA databases. **Tables 1** and **2** list the top 10 targets based on the two databases, respectively.

**Table 1:** Target genes of gigantol by Swiss Target Prediction.

Target	Gene name	UniProt ID	ChEMBL ID	Target class	Probability
Calmodulin	*CALM1*	P62158	CHEMBL6093	Unclassified protein	0.35446497
Cytochrome P450 19A1	*CYP19A1*	P11511	CHEMBL1978	Cytochrome P450	0.14973259
Arachidonate 15-lipoxygenase	*ALOX15*	P16050	CHEMBL2903	Enzyme	0.14152209
Arachidonate 12-lipoxygenase	*ALOX12*	P18054	CHEMBL3687	Enzyme	0.10877097
Glucocorticoid receptor	*NR3C1*	P04150	CHEMBL2034	Nuclear receptor	0.10877097
Transthyretin	*TTR*	P02766	CHEMBL3194	Secreted protein	0.10877097
Insulin-like growth factor I receptor	*IGF1R*	P08069	CHEMBL1957	Kinase	0.1005789
HMG-CoA reductase	*HMGCR*	P04035	CHEMBL402	Oxidoreductase	0.1005789
Estrogen receptor alpha	*ESR1*	P03372	CHEMBL206	Nuclear receptor	0.1005789
Estrogen receptor beta	*ESR2*	Q92731	CHEMBL242	Nuclear receptor	0.1005789

**Table 2:** Target genes of 13 bioactive components in gigantol by SEA.

Target key	Target name	Description	*p*-value	MaxTC
NU1M_BOVIN	MT-ND1	NADH-ubiquinone oxidoreductase chain 1	1.453E-51	0.34
TAAR5_HUMAN	TAAR5	Trace amine-associated receptor 5	1.389E-44	0.35
TBB1_HUMAN	TUBB1	Tubulin beta-1 chain	1.433E-44	0.52
Q6UCJ9_TOXGO	ENR	Enoyl-acyl carrier reductase	5.828E-40	0.42
SCMU_MYCTU		Secreted chorismate mutase	5.953E-37	0.29
DHCR7_HUMAN	DHCR7	7-dehydrocholesterol reductase	8.799E-37	0.4
CHMU_MYCTU		Intracellular chorismate mutase	3.875E-36	0.29
M9TGV3_MYCTX	inhA	Enoyl-[acyl-carrier-protein] reductase [NADH]	4.357E-35	0.37
INHA_MYCTU	inhA	Enoyl-[acyl-carrier-protein] reductase [NADH]	1.885E-34	0.4
FABI_ECOLI	fabI	Enoyl-[acyl-carrier-protein] reductase [NADH] FabI	3.39E-32	0.54

### 3.2 PPI network of gigantol-related HUA target

**Figure 1:**
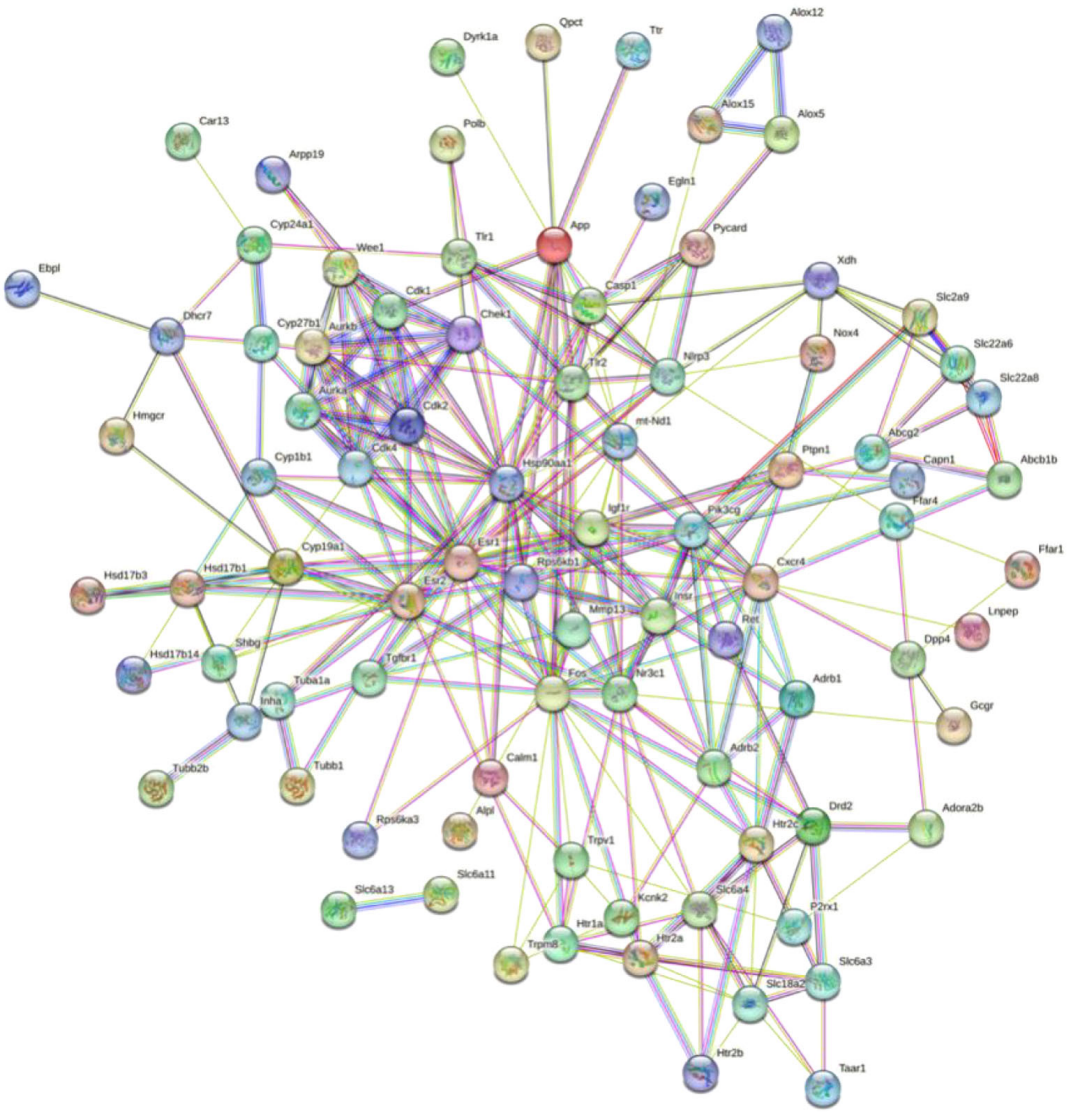
Construction and analysis of the HUA–gigantol PPI network.

The PPI network, which contains 101 nodes and 269 edges, was constructed by the STRING database, in which nodes represent proteins and edges stand for protein–protein interactions (**Figure 1**). Based on previous studies from our group, we selected *XDH, Casp1, Pycard, NLRP3, SLC6A4, SLC2A9, SLC18A2, SLC6A3*, and *SLC22A6* as hub genes. The correspondence between genes and proteins is shown in **Table 3**.

**Table 3:** Correspondence HUA-related genes with proteins.

Protein	120Gene
XOD	*XDH*
GLUT9	*SLC2A9*
OAT1	*SLC22A6*
OAT3	*SLC22A8*
ASC	*Pycard*
NLRP3	*NLRP3*
Caspase1	*Casp1*

### 3.3 Enrichment analysis

In order to elucidate the functions and the enriched pathways of the potential targets of gigantol, GO enrichment analysis and KEGG pathway enrichment analysis were carried out.

**Figure 2:**
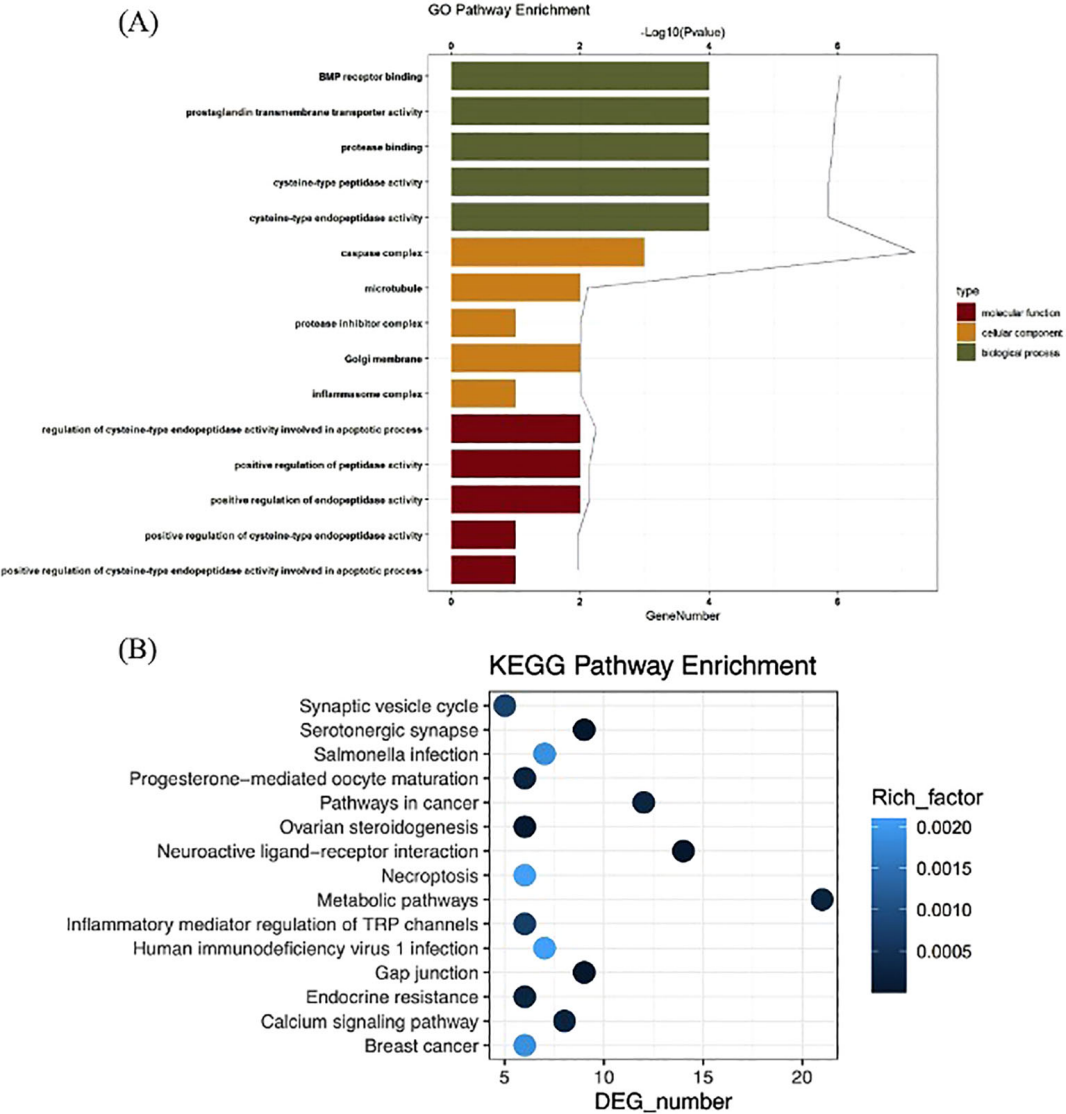
GO function analysis of gigantol core targets **(A)**. KEGG pathway enrichment analysis of gigantol core targets **(B)**.

The GO analysis consists of biological processes (BP), cellular component (CC), and molecular function (MF). In this study, a total of 398 significant GO terms, namely, 342 BP terms, 18 CC terms, and 38 MF terms, were obtained. The top eight significant enrichment terms of BP, CC, and MF with the highest gene counts were visualized in a histogram diagram in **Figure 2A**. The red color of a bar stands for the lower *q* value and the greater enrichment of the GO term. The results showed that the targets of gigantol for HUA were mainly enriched in response to positive regulation of cysteine-type endopeptidase activity involved in apoptotic process (GO:0043280), positive regulation of peptidase activity (GO:0010952), regulation of cysteine-type endopeptidase activity involved in apoptotic process (GO:0043281), and other biological processes; in the inflammasome complex (GO:0061702), membrane microdomain (GO:0098857), Golgi membrane (GO:0000139), and other cellular components; in cysteine-type endopeptidase activity involved in apoptotic process (GO:0097153), endopeptidase activity (GO:0004175), cysteine-type peptidase activity (GO:0008234), and other molecular functions.

The KEGG pathways were applied to explore the functions and signaling pathways of the targets of gigantol. There were 62 significant gigantol-HUA-related pathways, among them, the top 24 significant enrichment potential pathways with the highest gene counts were presented in a bar plot diagram (**Figure 2B**), indicating that gigantol plays an important role in the treatment of HUA through multiple targets and multiple pathways. The pathways with the top five highest gene counts were Metabolic pathways (*n* = 21), Neuroactive ligand–receptor interaction (*n* = 14), Pathways in cancer (*n* = 12), Serotonergic synapse (*n* = 9), and Gap junction (*n* = 9), which could be the key pathways in the effect of gigantol against HUA. Collectively, these results demonstrated that gigantol acts on HUA through multiple pathways, multiple targets, and overall cooperation.

### 3.4 Effects of gigantol on levels of UA, CRE, and BUN

**Figure 3:**
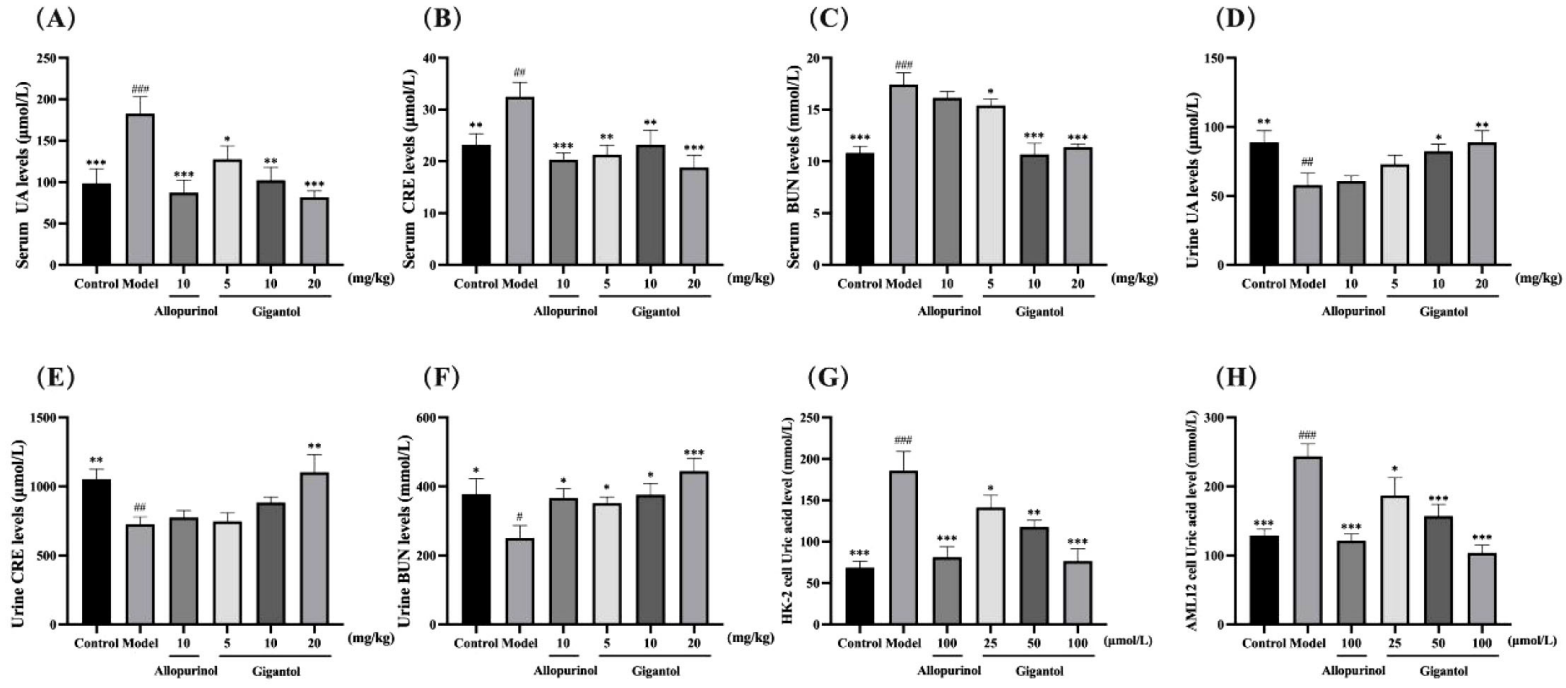
Therapeutic effects of gigantol on HUA models *in vivo* and *in vitro*. Effects of gigantol on serum UA **(A)** serum CRE **(B)** serum BUN **(C)** urine UA **(D)** urine CRE **(E)** and urine BUN **(F)** in HUA mice and UA levels in HK-2 **(G)** and AML12 **(H)** cells. Data were expressed as mean ± SEM; in **(A–C)** *n* = 8, in **(D–H)** *n* = 6. Compared with the control group, ^#^ *p* < 0.05, ^##^ *p* < 0.01, ^###^ *p* < 0.001. Compared with the model group, ^*^ *p* < 0.05, ^**^ *p* < 0.01, ^***^ *p* < 0.001.

**Figures 3A–F** show the effects of gigantol on serum and urine UA, CRE, and BUN in mice with HUA induced by potassium oxonate. Compared with the blank group, the levels of serum UA, CRE and BUN in the model mice significantly increased (UA and BUN: *p* < 0.001; CRE: *p* < 0.01), and the levels of urine UA, CRE, and BUN significantly decreased (UA and CRE: *p* < 0.01; BUN: *p* < 0.05). Thus, the potassium oxonate gavage-induced HUA model in mice was successfully established. Compared with the model group, each dose of gigantol significantly reduced the levels of serum UA, CRE, and BUN, especially at a high dose (*p* < 0.001). The content of UA in the urine of mice with HUA also increased significantly in a dose-dependent manner after the administration of gigantol (*p* < 0.01). The effects of gigantol on HUA cells are shown in **Figures 3G, H**. In HK-2 and AML12 cells, adenosine and xanthine can stimulate cells to produce UA, but abnormal elevation of UA can be suppressed by gigantol (*p* < 0.001). In conclusion, gigantol can reduce serum UA, CRE, and BUN levels while promoting the excretion of the above substances.

### 3.5 Effects of gigantol on XOD activity in the liver and AML12 cells with HUA

**Figure 4:**
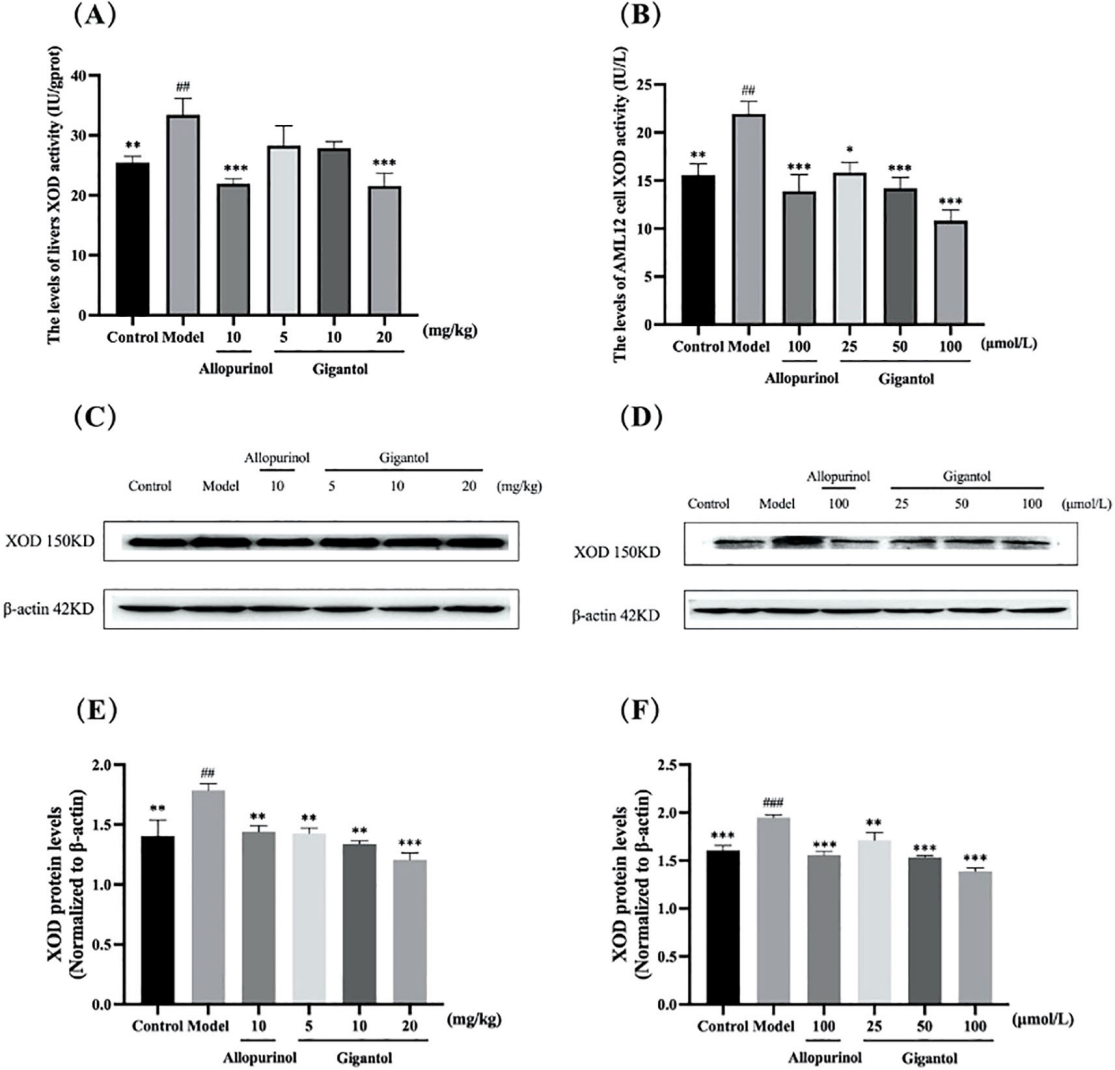
Effect of gigantol on XOD *in vivo* and *in vitro* models of HUA. Gigantol reduced XOD activity in mice liver **(A)** and AML12 cells **(B)**. Inhibition of protein expression of XOD in HUA mice liver **(C, E)** and AML12 cells **(D, F)**. Data were expressed as mean ± SEM; in **(A)**, *n* = 8; in **(B)**, *n* = 6; in **(E)** and **(F)**, *n* = 3. Compared with the control group, ^##^ *p* < 0.01, ^###^ *p* < 0.001. Compared with the model group, ^*^ *p* < 0.05, ^**^ *p* < 0.01, ^***^ *p* < 0.001.

Effects of gigantol on XOD activity in the liver (A) and AML12 (B) cells are shown in **Figure 4**. Compared with the normal group, the XOD levels in mice liver and AML12 cells of the model group significantly increased (*p* < 0.01); compared with the model group, allopurinol as a positive drug could significantly downregulate XOD levels (*p* < 0.001). Gigantol also downregulated XOD levels in AML12 cells (*p* < 0.05, *p* < 0.001, *p* < 0.001) in a dose-dependent manner. Notably, the effect of gigantol on XOD in the mice liver was weak; only the high dose inhibited XOD (*p* < 0.001), while the low and middle doses showed no significant difference compared with the model group (*p* > 0.05). Gigantol could inhibit UA production by inhibiting XOD viability, thereby reducing UA levels.

The changes of XOD protein expression by gigantol in HUA mice liver and AML12 cells are shown in **Figures 4C–F**. Compared with the normal control group, the expression of XOD protein in the liver of mice in the model group significantly increased (*p* < 0.01). Compared with the model group, the expression of XOD protein in the liver of mice treated with each dose of gigantol significantly decreased (*p* < 0.01, *p* < 0.01, *p* < 0.001). After treatment with gigantol, XOD protein expression significantly decreased in AML12 cells compared with the model group (*p* < 0.001).

### 3.6 Effects of gigantol on NLRP3 signal pathway

**Figure 5:**
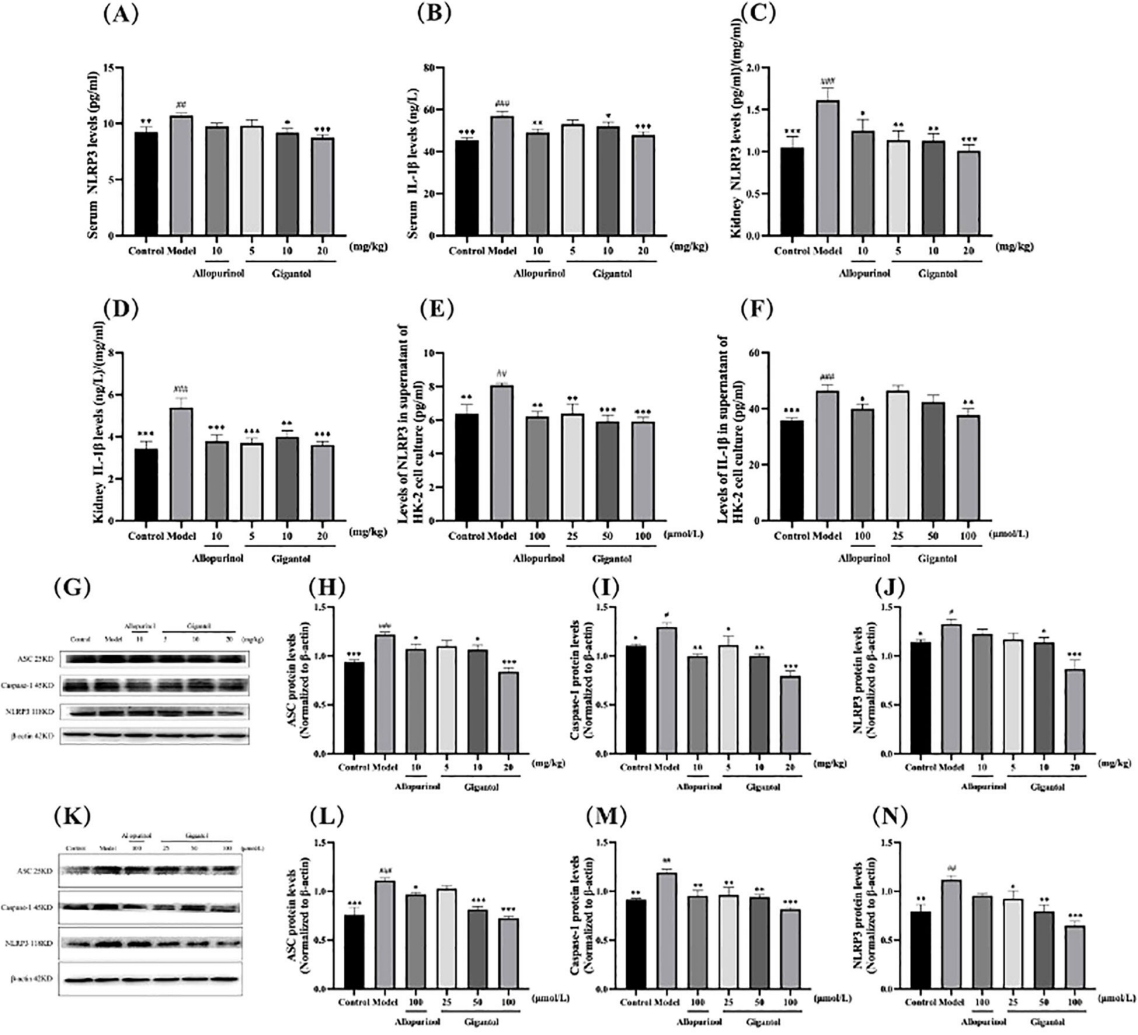
Effects of gigantol on the NLRP3 inflammatory signaling pathway. Gigantol decreased NLRP3 and IL-1β levels in serum **(A, B)**, kidney **(C, D)**, and HK-2 cells **(E, F)**. Gigantol decreased the expression of ASC, caspase-1, and NLRP3 in the kidney of HUA mice **(G–J)** and HK-2 cells **(K–N)**. Data are expressed as mean ± SEM; in **(A–D)**, *n* = 8; in **(E, F)**, *n* = 6; in **(G–N)**, *n* = 3. Compared with the control group, ^#^ *p* < 0.05, ^##^ *p* **<** 0.01, ^###^ *p* **<** 0.001. Compared with the model group, ^*^ *p* **<** 0.05, ^**^ *p* **<** 0.01, ^***^ *p* **<** 0.001.

The effects of gigantol on NLRP3 and IL-1β contents in HUA mice and HK-2 cells are shown in **Figures 5A–F**. NLRP3 and IL-1β contents in serum and kidney of mice in the model group significantly increased compared with those in the normal group (serum NLRP3: *p* < 0.01; all the rest: *p* < 0.001). After gigantol intragastric administration, NLRP3 and IL-1β levels decreased in low, medium, and high gigantol dose groups. Compared with the model group, the high dose group showed the most significant downward trend (*p* < 0.001).

We also detected the expression of NLRP3 signaling pathway-related proteins in mouse kidney (**Figures 5G–J**). Compared with the normal group, the expression of ASC, NLRP3, and caspase-1 proteins in the kidney of the model group was significantly increased (ASC: *p* < 0.001; NLRP3 and caspase-1: *p* < 0.05). Compared with the model group, the expression levels of ASC, NLRP3, and caspase-1 proteins in the gigantol high-dose group decreased significantly (ASC and caspase-1: *p* < 0.001; NLRP3: *p* < 0.01).

As shown in **Figures 5K–N**, gigantol significantly reduced NLRP3 in HK-2 cells in a dose-dependent manner. The inhibitory effect of gigantol on IL-1β was relatively weak, with only the high-dose group showing a downward trend (*p* < 0.01). The expression of three proteins related to the NLRP3 signaling pathway in the model group increased significantly compared with the blank group (ASC: *p* < 0.001; NLRP3 and caspase-1: *p* < 0.01). After the gigantol intervention, the protein levels decreased significantly, especially in the high-dose group (*p* < 0.001).

### 3.7 Effects of gigantol on TNF-α and IL-6 inflammatory factors in HUA mice and cells

**Figure 6:**
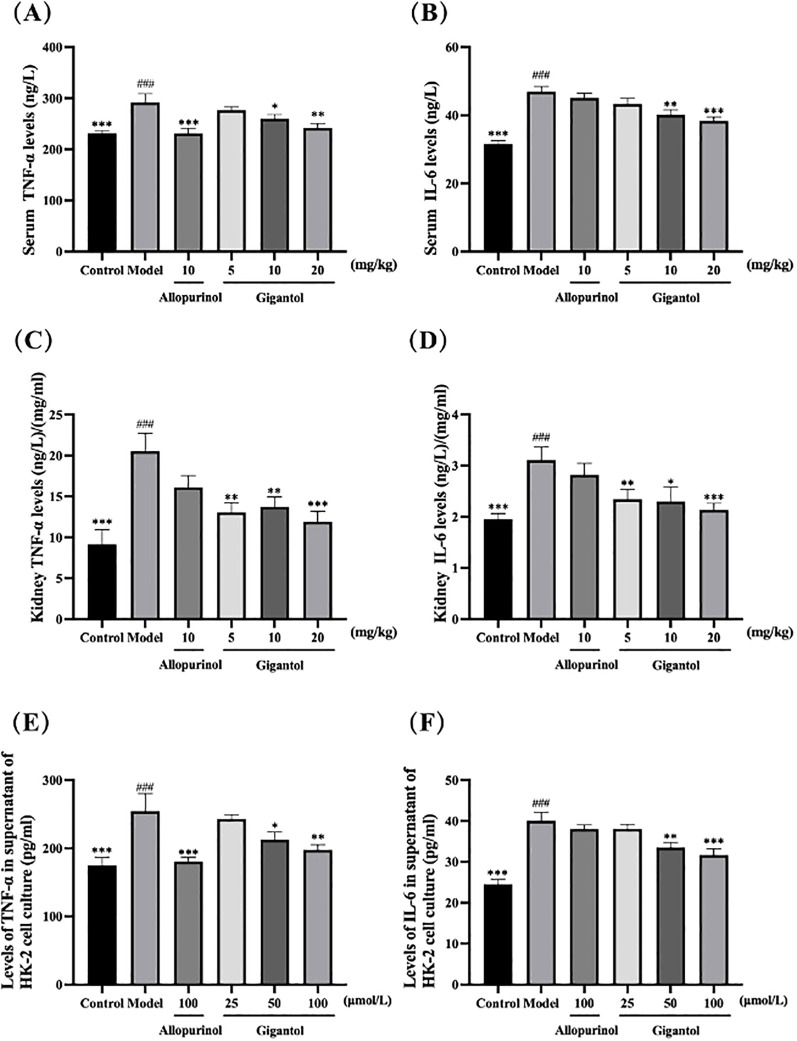
Effects of gigantol on TNF-α and IL-6 levels in HUA mice serum **(A, B)**, kidney **(C, D)**, and HK-2 cells **(E, F)**. Data were expressed as mean ± SEM; in **(A, B)**, *n* = 8; in **(C–F)**, *n* = 6. Compared with the control group, ^###^ *p* < 0.001. Compared with the model group, ^*^ *p* < 0.05, ^**^ *p* < 0.01, ^***^ *p* < 0.001.

Compared with the blank group, TNF-α and IL-6 levels in the serum and kidneys of mice in the model group were significantly higher (*p* **<** 0.001). Compared with the model group, serum and renal TNF-α and IL-6 levels in mice treated with gigantol were significantly lower (*p* **<** 0.01). Compared with the positive drug control group, TNF-α and IL-6 levels in the high dose group of gigantol were a little lower (**Figures 6A–D**).

We observed the same trend in HK-2 cells (**Figures 6E, F**). HK-2 cells subjected to adenosine and XOD induction had significantly higher levels of TNF-α and IL-6 compared with the blank control (*p* **<** 0.001). After gigantol treatment, the levels of inflammatory factors in HK-2 cells tended to decrease dose-dependently.

### 3.8 Effects of gigantol on the expression of UA transporters

**Figure 7:**
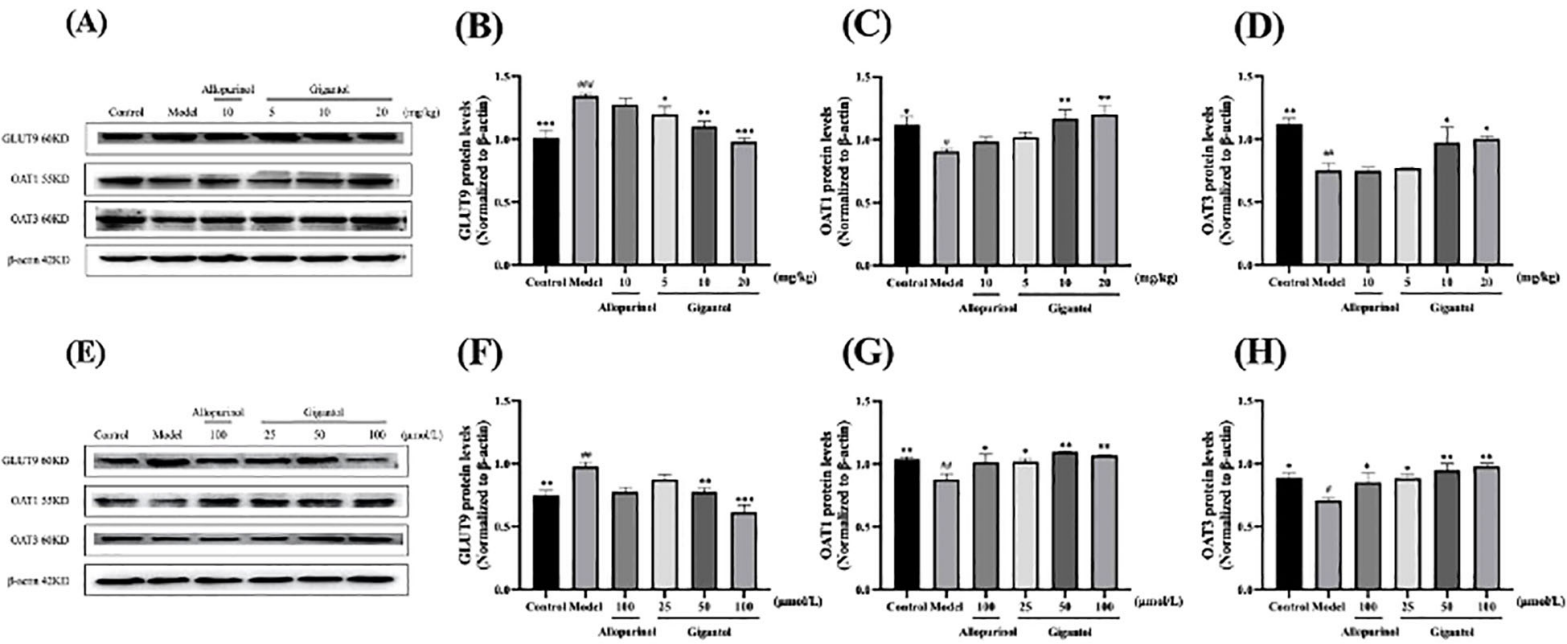
Effect of gigantol on UA transporters in *in vivo* and *in vitro* models of HUA. Gigantol inhibited GLUT9 protein expression and upregulated OAT1 and OAT3 protein expression in HUA mice **(A–D)** and HK-2 cells **(E–H)**. Data were expressed as mean ± SEM; *n* = 3. Compared with the control group, ^#^ *p* < 0.05, ^##^ *p* < 0.01, ^###^ *p* < 0.001. Compared with the model group, ^*^ *p* < 0.05, ^**^ *p* < 0.01, ^***^ *p* < 0.001.

**Figure 7** shows the effects of gigantol on UA transporters in HK-2 cells and in kidneys of HUA mice. Compared with the normal group, the GLUT9 protein expression level of the model mice significantly increased (*p* < 0.001), and OAT1 and OAT3 levels significantly decreased (OAT1: *p* < 0.05; OAT3: *p* < 0.01). Compared with the model group, gigantol significantly reduced GLUT9 protein expression in a dose-dependent manner. Gigantol only increased protein expression of OAT1 and OAT3 in the medium-dose group and the high-dose group (OAT1: *p* < 0.01; OAT3: *p* < 0.05), but not in the low-dose group compared with the model group. However, allopurinol did not show significant effects on the above three proteins (*p* > 0.05).

HK-2 cells exposed to adenosine and XOD showed an increase in GLUT9 protein expression and a decrease in OAT1 and OAT3 protein expression (GLUT9 and OAT1: *p* < 0.01; OAT3: *p* < 0.05). Gigantol decreased GLUT9 protein expression in HK-2 cells, especially in the high-dose group (*p* < 0.001). Meanwhile, gigantol could increase OAT1 and OAT3 protein expression in HK-2 cells in a dose-dependent manner.

### 3.9 Pathological changes of renal and liver tissues in mice treated by gigantol

**Figure 8:**
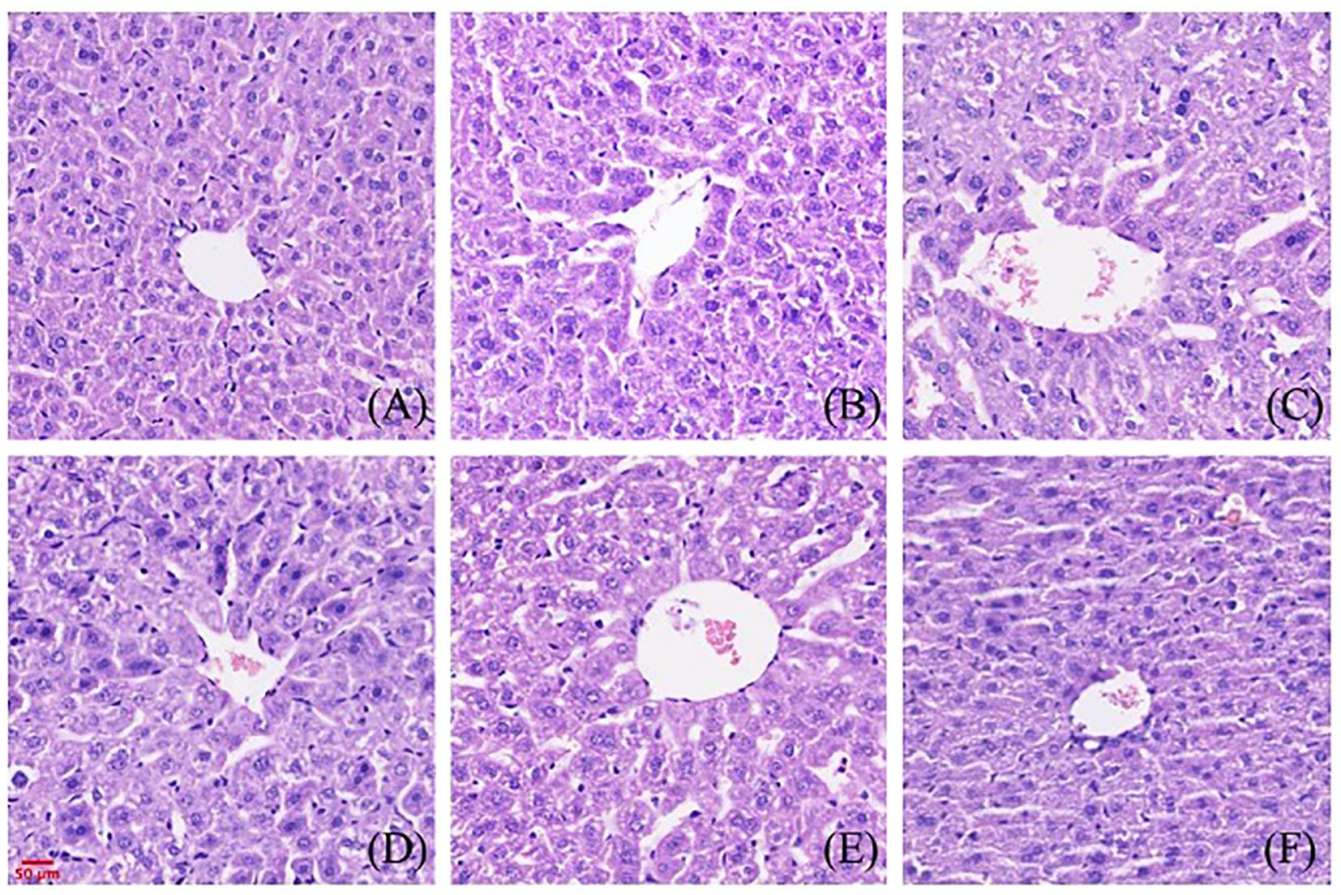
Effect of gigantol on pathological changes of liver tissue in mice with HUA (HE, 200×). **(A)** Blank control group. **(B)** Model control group. **(C)** Allopurinol group, 10 mg/kg. **(D)** Gigantol group, 5 mg/kg. **(E)** Gigantol group, 10 mg/kg. **(F)** Gigantol group, 20 mg/kg.

The effects of gigantol on liver histopathology in mice are shown in **Figure 8**. In the normal group, hepatocytes arranged in a cord shape, no degeneration and necrosis were observed, liver sinusoids showed no obvious expansion, and liver lobule structure was intact. There was no inflammatory cell infiltration and fibroplasia. In the model group, we observed hepatocyte degeneration with vacuole formation. The arrangement of hepatocytes was slightly disordered, and the cell boundary of hepatocytes was not clear. After treatment with gigantol, the atrophy of liver cells significantly improved, the arrangement of cells tended to be neat, and the structure of liver lobules was complete.

**Figure 9:**
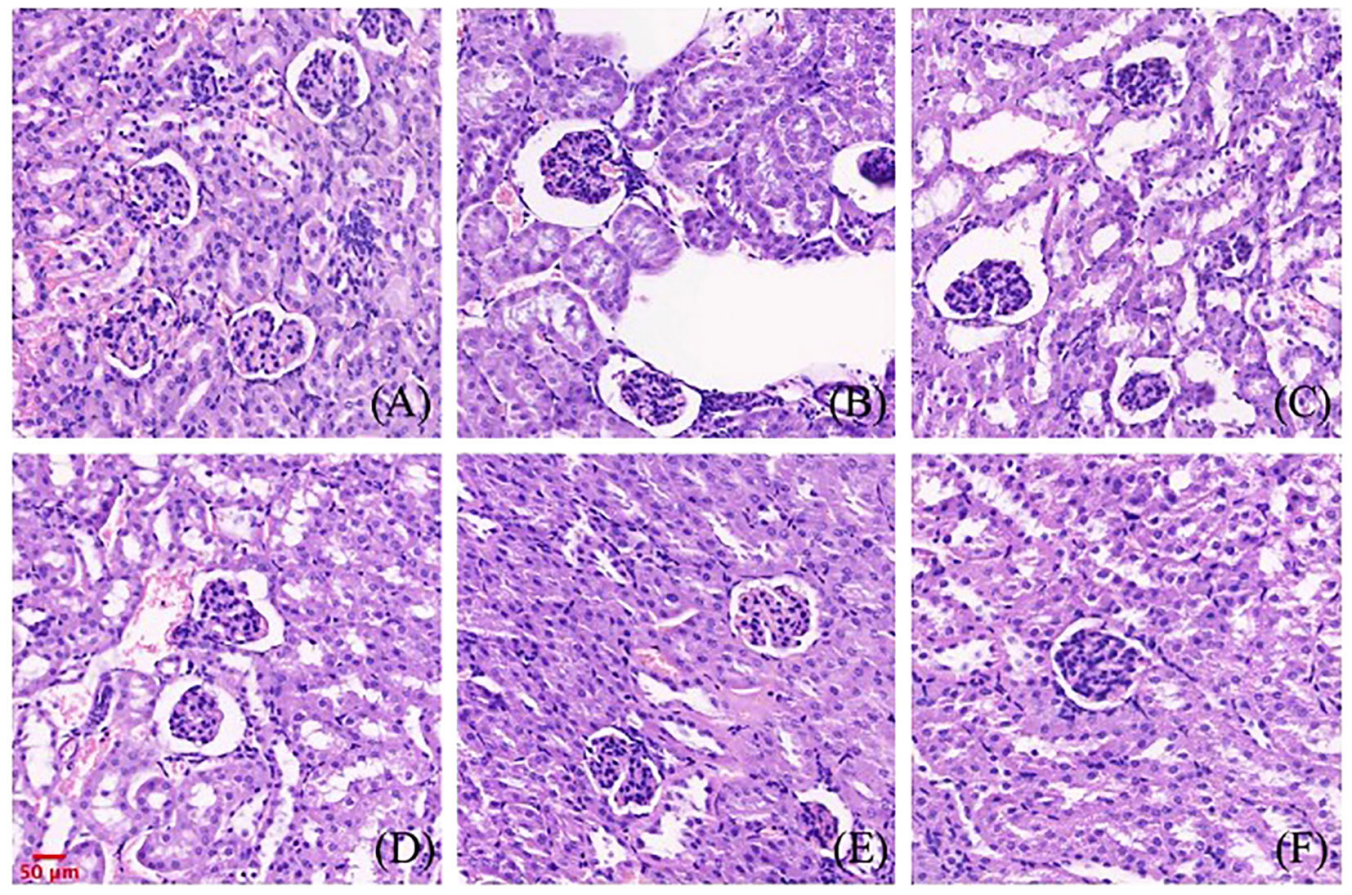
Effect of gigantol on pathological changes of renal tissue in mice with HUA (HE, 200×). **(A)** Blank control group. **(B)** Model control group. **(C)** Allopurinol group, 10 mg/kg. **(D)** Gigantol group, 5 mg/kg. **(E)**: Gigantol group, 10 mg/kg. **(F)** Gigantol group, 20 mg/kg.

The effects of gigantol on renal histopathology in mice are shown in **Figure 9**. Compared with the normal group, the renal tubules of the model group were dilated, and vacuolar degeneration of epithelial cells and a few brown urate crystals could be seen in the lumen. In the gigantol-treated groups, we found a trend of recovery in the injury of the glomerulus, and there were no urate crystals in the renal tubules.

### 3.10 Pharmacokinetics of gigantol in rats

**Figure 10:**
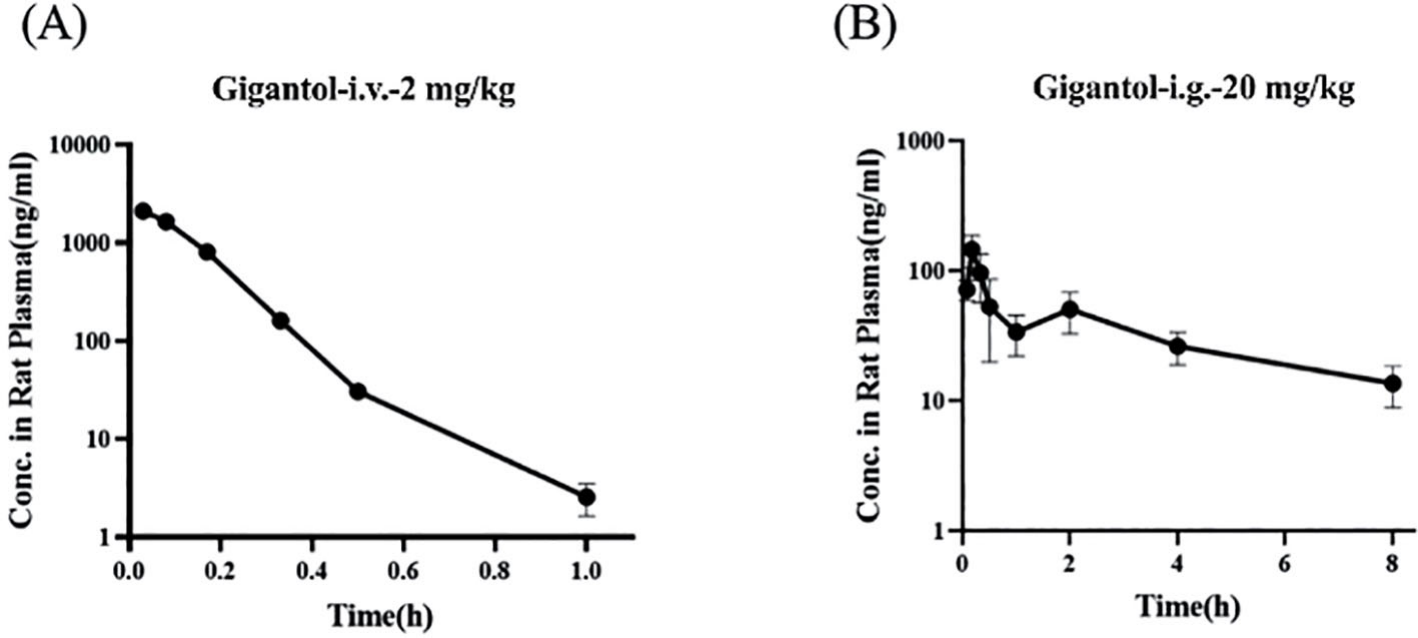
Blood concentration–time curves of gigantol in rats after intravenous **(A)** and gavage **(B)** administration.

**Figures 10A, B** show the blood concentration–time curves of gigantol in rats after intravenous and intragastric administration, respectively. The pharmacokinetic parameters of gigantol in rats after intravenous and gavage administration are shown in **Tables 4** and **5**. After intravenous administration, the half-life of gigantol was 0.10 ± 0.01 h, and the clearance rate was 5.30 ± 0.27 L/h/kg, indicating that the elimination of gigantol in the body was relatively fast, and rapid metabolism may be the main reason. After intragastric administration, the second peak appeared at approximately 2 h, suggesting liver–intestinal circulation of the drug *in vivo*. After oral administration, the *C*_max_ was approximately 145.81 ± 40.26 µg/L, and the *T*_max_ was approximately 0–1 h. The peak concentration was low and the peak time was short, suggesting that the drug might have intestinal metabolism, leading to the reduction of drug absorption into the blood circulation.

**Table 4:** Pharmacokinetic parameters of gigantol in rats after intravenous injection.

Parameter	Units	1	2	3	Average	SD
C_0_	µg/L	2,156.78	2,922.66	2,407.70	2,495.71	390.45
AUC_(0-t)_	h·µg/L	356.99	385.68	390.64	377.77	18.17
AUC_(0-∞)_	h·µg/L	357.38	385.88	391.13	378.13	18.16
t_1/2_	h	0.10	0.09	0.10	0.10	0.01
V	L/kg	0.80	0.67	0.74	0.74	0.07
CL	L/h/kg	5.60	5.18	5.11	5.30	0.27
MRT_(0-∞)_	h	0.12	0.11	0.12	0.12	0.01

**Table 5:** Pharmacokinetic parameters of gigantol in rats after gavage injection.

Parameter	Units	1	2	3	4	Average	SD
*C*_max_	µg/L	151.31	199.70	125.19	107.03	145.81	40.26
*T*_max_	h	0.17	0.17	0.17	0.17	0.17	0.00
AUC_(0-t)_	h·µg/L	277.17	369.00	217.51	138.31	250.50	97.34
AUC_(0-∞)_	h·µg/L	303.16	452.32	321.01	195.66	318.04	105.26
*t*_1/2_	h	2.12	3.21	5.00	2.37	3.18	1.30
V/F	L/kg	201.66	204.88	449.03	349.10	301.17	120.19
CL/F	L/h/kg	65.97	44.22	62.30	102.22	68.68	24.30
MRT_(0-∞)_	h	3.25	4.49	7.00	3.27	4.50	1.76
*F*	%	16.82					

### 3.11 Pharmacokinetics of allopurinol in rats

**Figure 11:**
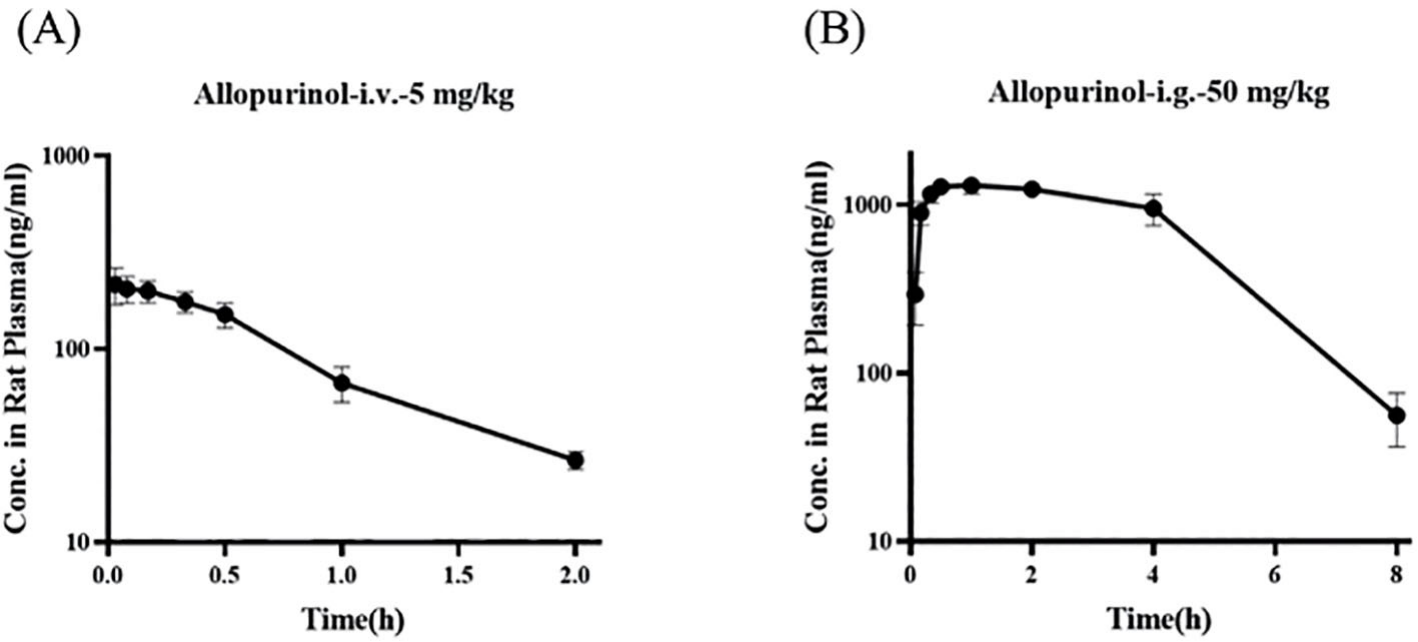
Blood concentration–time curves of allopurinol in rats after intravenous **(A)** and gavage administration **(B)**.

**Figures 11A, B** show the blood concentration–time curves of allopurinol in rats after intravenous and intragastric administration, respectively. The pharmacokinetic parameters of allopurinol in rats after intravenous and gavage administration are shown in **Tables 6** and **7**. After intravenous injection of 5 mg/kg allopurinol, *C*_0_ was approximately 224.62 ± 56.36 μg/L, and the fitting PK parameters showed that the half-life was only 0.56 h. After an intragastric administration of 50 mg/kg, *C*_max_ was approximately 1,353.33 ± 75.06 μg/L, *T*_max_ was approximately 1–2 h, AUC_(0-∞)_ was approximately 6,575.27 ± 301.97 h·μg/L, and absolute bioavailability was approximately 314.35%. These results suggest that the drug may undergo rapid metabolism after entering the blood or rapidly reach the tissues, resulting in low *C*_max_ values. Drugs were eliminated faster in the body, possibly because of faster metabolism or faster excretion.

**Table 6:** Pharmacokinetic parameters of allopurinol in rats after intravenous injection.

Parameter	Units	1	2	3	Average	SD
*C*_0_	µg/L	169.00	223.22	281.70	224.64	56.36
AUC_(0-t)_	h·µg/L	126.68	186.21	225.15	179.35	49.59
AUC_(0-∞)_	h·µg/L	170.08	207.21	250.23	209.17	40.11
*t*_1/2_	h	0.49	0.59	0.61	0.56	0.07
*V*	L/kg	20.68	20.60	17.58	19.62	1.76
CL	L/h/kg	29.40	24.13	19.98	24.50	4.72
MRT_(0-∞)_	h	0.73	0.81	0.83	0.79	0.05

**Table 7:** Pharmacokinetic parameters of allopurinol in rats after gavage injection.

Parameter	Units	1	2	3	Average	SD
*C*_max_	µg/L	1,310.00	1,440.00	1,310.00	1,353.33	75.06
*T*_max_	h	2.00	1.00	1.00	1.33	0.58
AUC_(0-t)_	h·µg/L	6,292.43	6,239.00	6,870.26	6,467.23	350.05
AUC_(0-∞)_	h·µg/L	6,428.54	6,374.71	6,922.57	6,575.27	301.97
*t*_1/2_	h	1.40	1.39	1.09	1.29	0.18
*V*/*F*	L/kg	15.75	15.77	11.31	14.28	2.57
CL/F	L/h/kg	7.78	7.84	7.22	7.61	0.34
MRT(0-∞)	h	2.06	2.68	2.75	2.50	0.38
*F*	%	314.35				

### 3.12 Molecular docking

**Figure 12:**
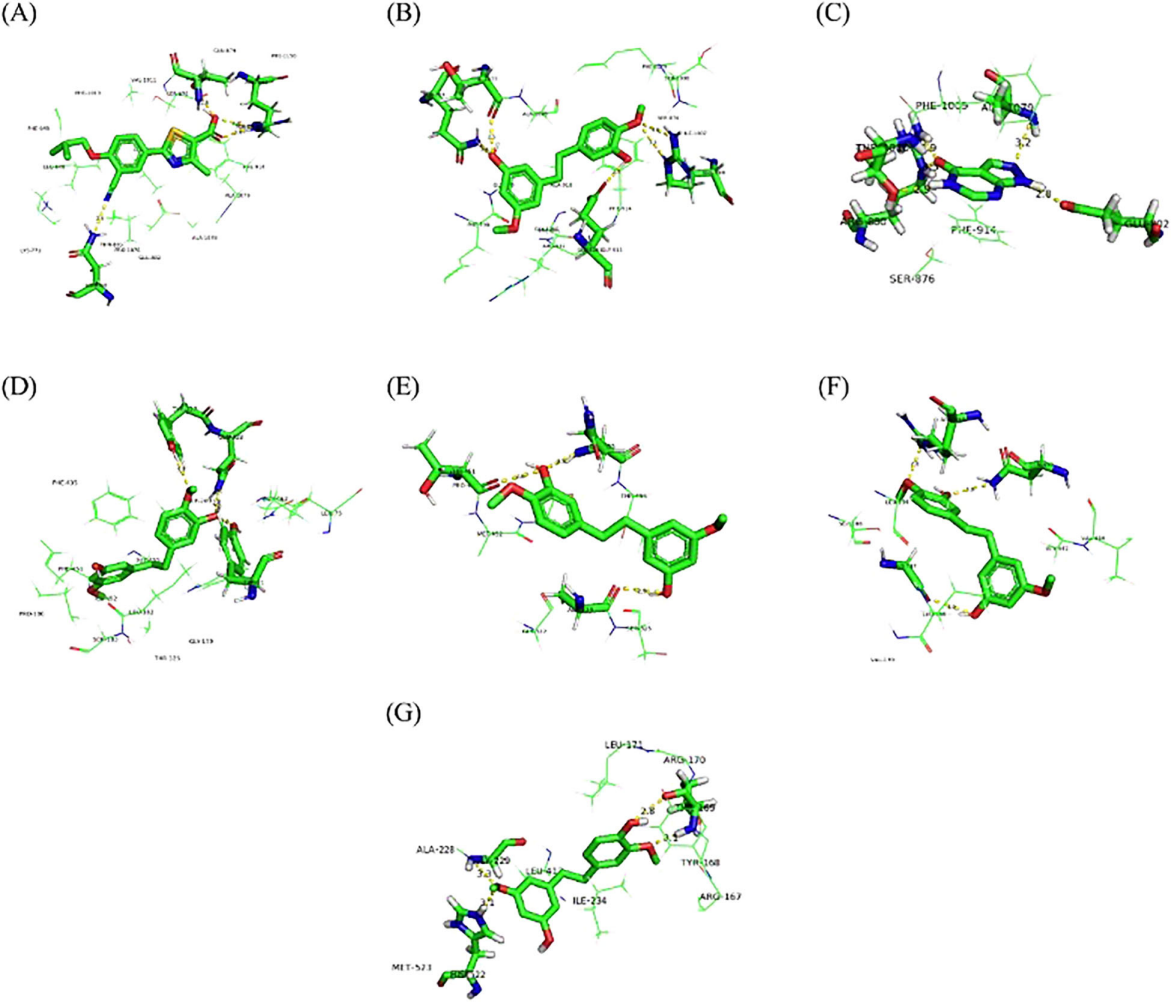
Molecular docking of febuxostat with XOD **(A)**, gigantol with XOD **(B)**, allopurinol with XOD **(C)**, gigantol with GLUT9 **(D)**, OAT1 **(E)**, OAT3 **(F)**, and NLRP3 **(G)**.

To ensure the accuracy of the docking method, we docked febuxostat, the original ligand of XOD. The binding energy of febuxostat to protein was −8.94 kcal/mol (**Figure 12A**). The cyano group and the carboxyl group on the thiazole ring in the structure of febuxostat formed hydrogen bonds with amino acid residues Asn768, Thr1010, and Arg880, with bond lengths of 2.5, 2.8, 3.24, and 2.64Å, respectively. The thiazole ring was involved in a π–π interaction with phe-914, and the docking site was consistent with a previous report (24), demonstrating the accuracy of the present docking method.

**Figure 12B** shows that gigantol had a binding energy of −8.78 kcal/mol with XOD, The aromatic ring of gigantol formed hydrogen bonds with amino acid residues Arg880, Glu1261, Gln767, and Thr1077, respectively. The bond lengths were 3.0, 3.26, 2.75, 2.64, and 2.53 Å, respectively. π–π interaction occurred between the aromatic ring and Phe914, and the docking site was consistent with the original ligand.

**Figure 12C** shows that the binding capacity of allopurinol and XOD was −5.97 kcal/mol, and allopurinol formed hydrogen bonding with amino acid residues Glu802, Arg880, Thr1010, and Ala1079. The bond lengths were 2.82, 2.85, 3.27, 2.93, and 3.19 Å, respectively. π–π interaction took place between pyrimidine ring in the allopurinol structure and amino acid residue Phe914, and the docking site was consistent with a previous report (24, 30).

**Figure 12D** shows that the binding energy of gigantol to hGLUT9 was −5.45 kcal/mol. The hydroxyl group and methoxy group on the aromatic ring formed hydrogen bonds with amino acid residues Tyr327, Tyr71, and Gln328, with bond lengths of 3.11, 2.51, and 2.75 Å, respectively, and the docking sites were consistent with those reported previously (28), indicating that the compound dendrobium could interact with hGLUT9.

**Figure 12E** shows that the binding energy of gigantol and OAT1 was −5.79 kcal/mol. The hydroxyl group and methoxy group on the aromatic ring formed hydrogen bonds with amino acid residues Gln455, Thr451, and Ala324, respectively. The docking sites were consistent with those reported previously (29), indicating that the compound dendrobium could interact with OAT1.

**Figure 12F** shows that the binding energy of gigantol and OAT3 was −5.72 kcal/mol. The hydroxyl group and methoxy group on the aromatic ring structure of the compound formed hydrogen bonds with amino acid residues Asn450, Arg454, and Gly137, with bond lengths of 3.02, 2.60, and 2.96 Å, respectively. The docking sites were consistent with those reported previously (29), indicating that the compound dendrobium could interact with OAT3.

**Figure 12G** shows that the binding energy of NLRP3 and gigantol was −7.85 kcal/mol. The hydroxyl group and methoxy group on the aromatic ring structure formed hydrogen bonds with amino acid residues Thr169, Gly229, and His522, with bond lengths of 2.79, 3.10, 3.28, and 3.10 Å, respectively. The docking sites were consistent with those reported previously (25, 31), indicating that the compound dendrobiol can interact with NLRP3.

Molecular docking revealed distinct binding characteristics among the three drugs. Febuxostat showed the strongest XOD affinity (−8.94 kcal/mol) through rigid hydrogen bonds with Asn768/Thr1010/Arg880. Gigantol exhibited comparable binding (−8.78 kcal/mol, Δ= 0.16 kcal/mol) with additional hydrogen bonds (Arg880/Glu1261/Gln767/Thr1077) via its flexible hydroxyl/methoxy groups, demonstrating superior adaptability. In contrast, allopurinol had significantly weaker binding (−5.97 kcal/mol) and requires metabolic activation.

Gigantol uniquely combines febuxostat-like π-π stacking (Phe914) with an extended hydrogen-bonding network, suggesting both high potency and improved selectivity potential—key advantages for next-generation urate-lowering drug development.

## 4 Discussion

HUA can lead to a series of diseases, including gout, kidney stones, hypertension, and cardiovascular diseases. Epidemiological studies have demonstrated that the incidence of HUA is steadily increasing, and its prevention and control pose significant challenges (32). HUA is closely related to excessive UA production and reduced UA excretion (33). At present, the regulation of blood UA levels is achieved through two primary mechanisms: one is the inhibition of UA production. XOD is an enzyme necessary for the conversion of hypoxanthine to UA. An effective way to treat gout is to inhibit the activity of XOD and thus inhibiting the formation of UA. In recent years, allopurinol and febuxostat are the main drugs for HUA, but these drugs have adverse reactions such as nausea and vomiting, which limit their clinical use (34). Although these drugs have significant efficacy, the hypersensitivity syndrome caused by these drugs can affect the recovery of patients (35, 36). Second, HUA can be treated by promoting the excretion of UA. Drugs that promote the excretion of UA include probenecid and benzbromarone. However, probenecid and benzbromarone have side effects such as gastrointestinal reaction, renal colic, and triggering acute gout attack (37), which limit their clinical application. Therefore, novel anti-gout and HUA drugs with high efficiency and low toxicity are urgently needed.

*Dendrobium officinale* mainly contains polysaccharides, astragalus, phenols, lignin, and other compounds, which have anti-aging, antitumor, hypoglycemic, and immune-enhancing effects (38). Our previous studies have found that the extract of *D. officinale* could inhibit XOD, could lower the level of UA, and exhibited therapeutic efficacy on HUA. Gigantol was extracted from *D. officinale*, and we hypothesize that it may have a therapeutic effect on HUA.

In this study, a HUA mouse model was established by gavage of potassium oxonate. With allopurinol as a positive control, the effects of different doses of gigantol on UA, CRE, and BUN in HUA mice were compared. We found that gigantol significantly decreased serum UA levels and increased urine UA levels in HUA mice. At the same time, gigantol reduced serum CRE and BUN, and promoted the excretion of these two substances. Combined with the pathological analysis of the liver and kidney, we speculated that gigantol can ameliorate liver and kidney tissue damage caused by excessive UA.

Next, we used network pharmacology methods to explore the pharmacological mechanism by which gigantol reduces UA and to identify potential targets of gigantol in the treatment of HUA. Our results suggest that gigantol may act on XOD, the NLRP3 inflammatory pathway, and GLUT9 and other UA transporters through targeting *XDH, SLC2A9, SLC22A6, SLC22A8, Pycard, NLRP3*, and *Casp1*.

Excessive UA production is related to enhanced activity of XOD, the key enzyme regulating UA production (39). XOD is the rate-limiting enzyme that converts purines to UA during UA production (40). XOD facilitates purine metabolism and converts hypoxanthine and xanthine into UA (41, 42). Therefore, inhibition of XOD activity is effective in controlling UA levels. We examined the effects of gigantol on XOD activity and expression in mice liver and AML12 cells. The results indicate that gigantol can significantly inhibit XOD activity, thereby reducing UA production and serum UA level.

UA crystals in the body of patients with HUA act as an activation signal, which is recognized and bound by the NLRP3 leucine-rich repeats to activate NLRP3. Activated NLRP3 recruits ASC and caspase-1 to form the NLRP3 inflammasome, which regulates the activity of caspase-1 and cleaves IL-1β precursor protein to become mature IL-1β, ultimately promoting inflammation. In the kidneys of model mice, protein expression of NLRP3, ASC, and caspase-1 increased, along with serum levels of inflammatory factors such as NLRP3, IL-1β, and TNF-α, indicating that the inflammation reaction resulting from the aberrant expression of NLRP3, ASC, and caspase-1 in HUA mice may be related to excessive serum UA level. After gigantol administration, the levels of inflammatory factors in serum and kidney tissues of HUA mice decreased, and protein expressions of NLRP3, ASC, and caspase-1 in kidney tissues were significantly downregulated, suggesting that gigantol inhibits NLRP3 signaling pathway in HUA mice. We further validated that gigantol could inhibit the NLRP3 signaling pathway in HK-2 cells. The mechanism may be that gigantol inhibits the activities of NLRP3, ASC, and caspase-1.

Renal UA excretion is closely related to a variety of UA transporters such as OAT1, OAT3, and GLUT9. As a member of the glucose transporter family, GLUT9 can accelerate the reabsorption of UA by transporting glucose (43). OAT1 and OAT3 are key proteins involved in UA secretion and are distributed in the basolateral aspect of proximal tubular epithelial cells. OAT1 and OAT3 act as an organic anion/ketoglutarate exchanger, removing α-ketoglutarate while completing UA uptake and reducing the content of UA in the blood (44). The results of this study showed that gigantol at different doses could significantly upregulate the expression of OAT1 and OAT3 while inhibiting the expression of GLUT9 in the kidney of HUA mice and in HK-2 cells, reducing the reabsorption of UA by proximal tubular epithelial cells, and increasing the excretion of UA, so as to improve HUA. By molecular docking experiments, we further verified that gigantol could interact with the above-mentioned UA transporters.

Despite its efficacy in HUA models, gigantol’s clinical translation faces challenges due to low oral bioavailability and rapid clearance. Future studies should prioritize selecting appropriate doses to address these questions. According to related studies, nano-formulation may enhance gigantol’s solubility and prolong circulation time. Nano-formulations (e.g., polymeric nanoparticles and liposomes) can address gigantol’s poor solubility and rapid clearance by enhancing dissolution and prolonging circulation. If successful, gigantol can be a promising candidate for HUA treatment.

## 5 Conclusions

**Figure 13:**
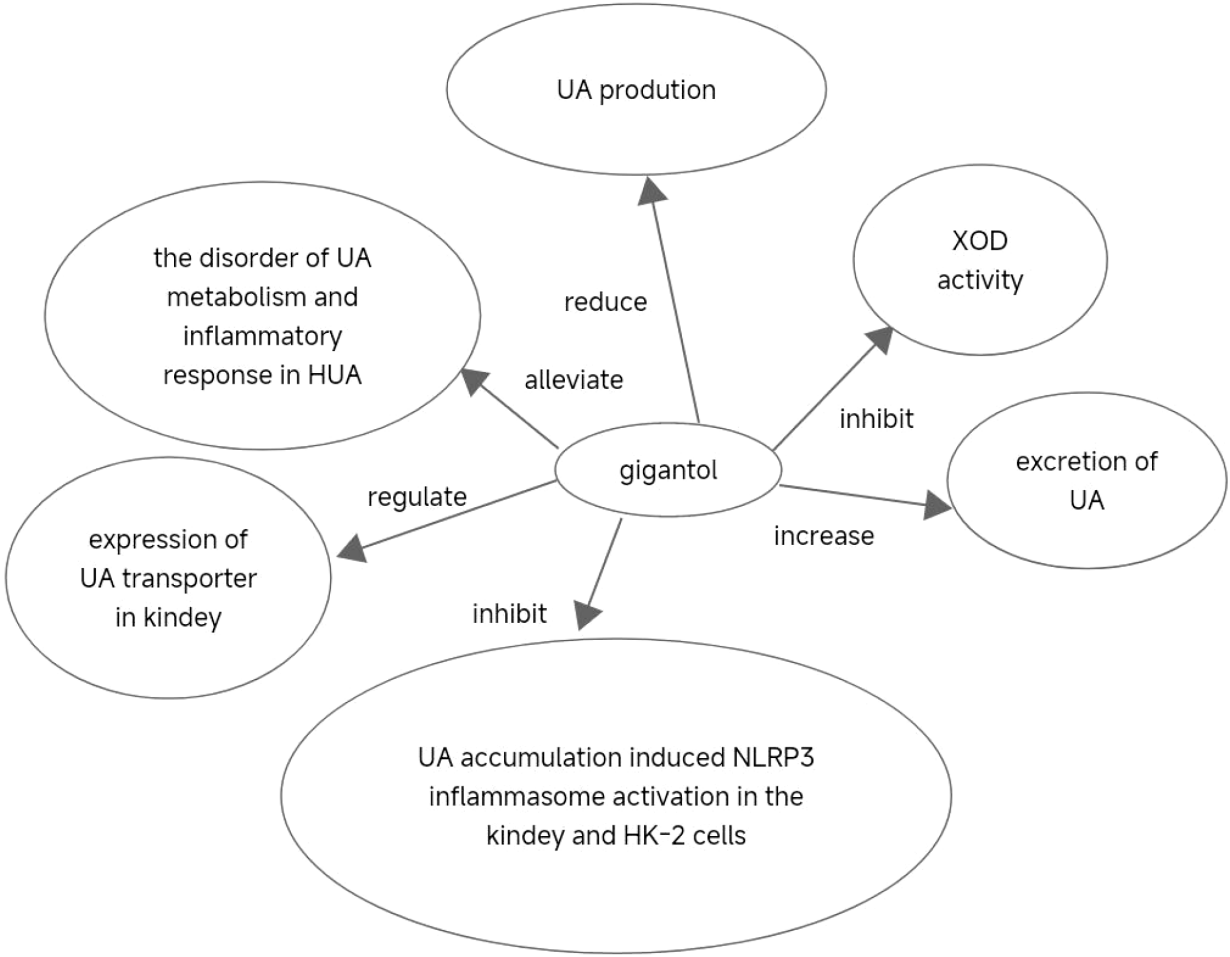
Translation implications and clinical development of gigantol.

In conclusion, our findings suggest that gigantol could alleviate the imbalance of UA metabolism and inflammatory response in HUA, and exerts a therapeutic effect on HUA. The beneficial effect of gigantol may be achieved by inhibiting XOD activity and reducing UA production. In addition, gigantol could increase the excretion of UA and reduce the content of UA by regulating the expression of UA transporter in the kidney. Furthermore, gigantol could inhibit UA accumulation-induced NLRP3 inflammasome activation in the kidney and HK-2 cells, thereby reducing renal inflammation. Therefore, our study provides support for the development of a new generation of anti-HUA agents with gigantol as a core component (**Figure 13**).

## Data Availability

The raw data supporting the conclusions of this article will be made available by the authors, without undue reservation.

## Supplementary material

Supplementary Image 1 The ASC protein levels in HK-2 cells are shown. Lane 1: control group; Lane 2: model group; Lane 3: 100 μmol/L allopurinol treatment; Lanes 4–6: 25, 50, and 100 µmol/L gigantol treatment, respectively. ASC was significantly reduced in the 50 and 100 µmol/L gigantol groups compared to the model group, indicating that gigantol suppresses inflammation by downregulating ASC, a key protein in the NLRP3 signaling pathway.

Supplementary Image 2 The Caspase-1 protein levels in HK-2 cells are shown. Lane assignments are as in Figure 1. Caspase-1 was significantly reduced at all gigantol concentrations (25, 50, and 100 µmol/L) compared to the model group, demonstrating that gigantol inhibits inflammation by suppressing Caspase-1, another NLRP3 pathway-related protein.

Supplementary Image 3 The GLUT9 protein levels in HK-2 cells are shown. Lane assignments are as in Figure 1. GLUT9 was significantly decreased, particularly at 100 µmol/L gigantol, compared to the model group, suggesting that gigantol inhibits uric acid (UA) transport by downregulating GLUT9 expression.

Supplementary Image 4 The NLRP3 protein levels in HK-2 cells are shown. Lane assignments are as in Figure 1. NLRP3 was moderately reduced at 25 μmol/L and significantly reduced at 50 and 100 µmol/L gigantol compared to the model group, confirming that gigantol attenuates inflammation by suppressing NLRP3, a central component of the NLRP3 inflammasome pathway.

Supplementary Image 5 Shows the OAT1 protein levels in HK-2 cells. The first column represents the control group, the second column the model group, and the third column the treatment with 100 µmol/L allopurinol. The fourth, fifth, and sixth columns represent the treatments with 25, 50, and 100 µmol/L gigatol, respectively. Under the treatments of 50 and 100 µmol/L gigatol, the OAT1 protein levels were significantly increased compared with the model group, indicating that gigatol can increase the expression of OAT1 protein in a dose-dependent manner.

Supplementary Image 6 Shows the OAT3 protein levels in HK-2 cells. The first column represents the control group, the second column the model group, and the third column the treatment with 100 µmol/L allopurinol. The fourth, fifth, and sixth columns represent the treatments with 25, 50, and 100 µmol/L gigatol, respectively. Under the treatments of 50 and 100 µmol/L gigatol, the OAT3 protein levels were significantly increased compared with the model group, indicating that gigatol can increase the expression of OAT3 protein in a dose-dependent manner.

Supplementary Image 7 Shows the XOD levels in AML12 cells. The first column represents the control group, the second column the model group, and the third column the treatment with 100 µmol/L allopurinol. The fourth, fifth, and sixth columns represent the treatments with 25, 50, and 100 µmol/L gigatol, respectively. Compared with the model group, the XOD levels under gigatol treatment were significantly reduced in a dose-dependent manner.

Supplementary Image 8 Shows the Western blot result of the internal reference protein β-action.

Supplementary Table 1 The data presented in this Table regarding XOD represents the protein expression of XOD in AML12 cells, while β denotes the expression of the reference protein. Compared with the normal group, the XOD levels in AML12 cells of the model group significantly increased, compared with the model group, allopurinol as a positive drug could significantly downregulate XOD levels. Gigantol also downregulated XOD levels in AML12 cells in a dose-dependent manner. The ASC data reflects the protein expression of ASC in HK-2 cells, CASPASE indicates the protein expression of Caspase-1 in HK-2 cells, and NLRP3 signifies the protein expression of NLRP3 in HK-2 cells, with β representing the reference protein expression. The expression of three proteins related to NLRP3 signaling pathway in the model group increased significantly compared with the blank group. After the gigantol intervention, the protein levels decreased significantly, especially in the high-dose group. The GLUT9 data denotes the protein expression of GLUT9 in HK-2 cells, OAT1 represents the protein expression of OAT1 in HK-2 cells, and OAT3 indicates the protein expression of OAT3 in HK-2 cells. HK-2 cells exposed to adenosine and xanthine oxidase showed increase in GLUT9 protein expression and decrease in OAT1 and OAT3 protein expression. Gigatol decreased GLUT9 protein expression in HK-2 cells, especially in the high-dose group. Meanwhile, gigantol could increase OAT1 and OAT3 protein expression in HK-2 cells in a dose-dependent manner.
