# Supplementary figures and images for: Therapeutic effect and mechanism of gigantol on hyperuricemia
Source: Front Endocrinol (Lausanne). 2025 Jul 29;16:1474808. doi: 10.3389/fendo.2025.1474808 (PMC12339328; doi:10.3389/fendo.2025.1474808)

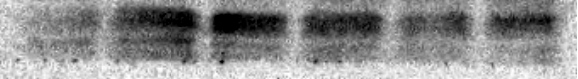

Supplement: Supplementary Image 1 — The ASC protein levels in HK-2 cells are shown. Lane 1: control group; Lane 2: model group; Lane 3: 100 μmol/L allopurinol treatment; Lanes 4–6: 25, 50, and 100 µmol/L gigantol treatment, respectively. ASC was significantly reduced in the 50 and 100 µmol/L gigantol groups compared to the model group, indicating that gigantol suppresses inflammation by downregulating ASC, a key protein in the NLRP3 signaling pathway. [file Image1.tif]

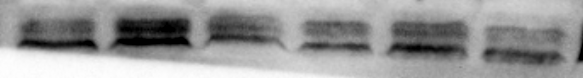

Supplement: Supplementary Image 2 — The Caspase-1 protein levels in HK-2 cells are shown. Lane assignments are as in Figure 1. Caspase-1 was significantly reduced at all gigantol concentrations (25, 50, and 100 µmol/L) compared to the model group, demonstrating that gigantol inhibits inflammation by suppressing Caspase-1, another NLRP3 pathway-related protein. [file Image2.tif]

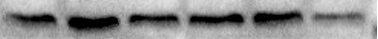

Supplement: Supplementary Image 3 — The GLUT9 protein levels in HK-2 cells are shown. Lane assignments are as in Figure 1. GLUT9 was significantly decreased, particularly at 100 µmol/L gigantol, compared to the model group, suggesting that gigantol inhibits uric acid (UA) transport by downregulating GLUT9 expression. [file Image3.tif]

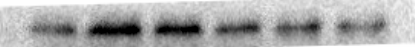

Supplement: Supplementary Image 4 — The NLRP3 protein levels in HK-2 cells are shown. Lane assignments are as in Figure 1. NLRP3 was moderately reduced at 25 μmol/L and significantly reduced at 50 and 100 µmol/L gigantol compared to the model group, confirming that gigantol attenuates inflammation by suppressing NLRP3, a central component of the NLRP3 inflammasome pathway. [file Image4.tif]

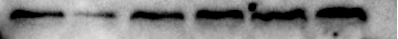

Supplement: Supplementary Image 5 — Shows the OAT1 protein levels in HK-2 cells. The first column represents the control group, the second column the model group, and the third column the treatment with 100 µmol/L allopurinol. The fourth, fifth, and sixth columns represent the treatments with 25, 50, and 100 µmol/L gigatol, respectively. Under the treatments of 50 and 100 µmol/L gigatol, the OAT1 protein levels were significantly increased compared with the model group, indicating that gigatol can increase the expression of OAT1 protein in a dose-dependent manner. [file Image5.tif]

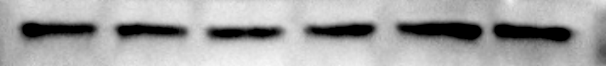

Supplement: Supplementary Image 6 — Shows the OAT3 protein levels in HK-2 cells. The first column represents the control group, the second column the model group, and the third column the treatment with 100 µmol/L allopurinol. The fourth, fifth, and sixth columns represent the treatments with 25, 50, and 100 µmol/L gigatol, respectively. Under the treatments of 50 and 100 µmol/L gigatol, the OAT3 protein levels were significantly increased compared with the model group, indicating that gigatol can increase the expression of OAT3 protein in a dose-dependent manner. [file Image6.tif]

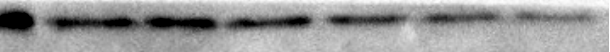

Supplement: Supplementary Image 7 — Shows the XOD levels in AML12 cells. The first column represents the control group, the second column the model group, and the third column the treatment with 100 µmol/L allopurinol. The fourth, fifth, and sixth columns represent the treatments with 25, 50, and 100 µmol/L gigatol, respectively. Compared with the model group, the XOD levels under gigatol treatment were significantly reduced in a dose-dependent manner. [file Image7.tif]

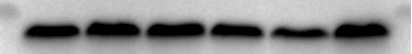

Supplement: Supplementary Image 8 — Shows the Western blot result of the internal reference protein β-action. [file Image8.tif]
